# Synthesis and Multiple Incorporations of 2′‐*O*‐Methyl‐5‐hydroxymethylcytidine, 5‐Hydroxymethylcytidine and 5‐Formylcytidine Monomers into RNA Oligonucleotides

**DOI:** 10.1002/cbic.201700492

**Published:** 2017-10-09

**Authors:** Arun A. Tanpure, Shankar Balasubramanian

**Affiliations:** ^1^ Department of Chemistry University of Cambridge Lensfield Road Cambridge CB2 1EW UK; ^2^ Cancer Research (UK) Cambridge Institute Li Ka Shing Centre University of Cambridge Robinson Way Cambridge CB2 0RE UK; ^3^ School of Clinical Medicine University of Cambridge Cambridge CB2 0SP UK

**Keywords:** epigenetics, formylcytosine, hydroxymethyl- cytosine, methylcytosine, RNA

## Abstract

The synthesis of 2′‐*O*‐methyl‐5‐hydroxymethylcytidine (hm^5^Cm), 5‐hydroxymethylcytidine (hm^5^C) and 5‐formylcytidine (f^5^C) phosphoramidite monomers has been developed. Optimisation of mild post‐synthetic deprotection conditions enabled the synthesis of RNA containing all four naturally occurring cytosine modifications (hm^5^Cm, hm^5^C, f^5^C plus 5‐methylcytosine). Given the considerable interest in RNA modifications and epitranscriptomics, the availability of synthetic monomers and RNAs containing these modifications will be valuable for elucidating their biological function(s).

Post‐transcriptional chemical modifications in RNA are more diverse and complex than epigenetic modifications in DNA and in histones. So far more than 140 chemically distinct RNA modifications have been identified in various species. The majority of these modifications involve methylation, of which 2′ ribose sugar methylation is the most abundant.^1^ These modifications were initially considered as static and stable marks; however, recent studies have revealed their dynamic nature and involvement in important gene‐regulatory functions.^2^ For instance, *N* ^6^‐methyladenosine (m^6^A), a predominant internal modification in eukaryotic messenger RNA (mRNA), can be oxidatively converted to adenosine by demethylases such as fat mass and obesity‐associated protein (FTO) and alkB homologue 5 (ALKBH5).^3^ This reversible adenosine methylation is proposed to be involved in RNA maturation, protein translation and gene expression.^4^ 5‐Methylcytidine (m^5^C) is another important methylated ribonucleoside that exists in transfer RNA (tRNAs), ribosomal RNA (rRNA), and in the untranslated regions of mRNAs.^2^, ^5^ We and others have demonstrated that m^5^C can be oxidatively metabolised to produce 5‐hydroxymethylcytidine (hm^5^C) and 5‐formylcytidine (f^5^C; Scheme 1).^6^, ^7^ Additionally, it is proposed that m^5^C is essential for mRNA export and post‐transcriptional regulation.^8^ Significant enrichment of hm^5^C in polyadenylated RNA compared to total RNA further suggests that biosynthesis of hm^5^C might be a part of a dynamic regulatory mechanism of RNA.6b, 9 The f^5^C modification is prevalent at the wobble position of an anticodon loop of mitochondrial methionine tRNA in many species, including humans. It was observed that f^5^C provides flexibility to the loop and affords an ability to decode both AUG and AUA in translational initiation and elongation sites of mRNA.^10^ Chemical labelling coupled with liquid chromatography‐mass spectrometry (LC‐MS) analysis revealed the existence of 5‐carboxycytidine (ca^5^C) in mouse liver tissue, albeit at a very low concentration.^11^ Recently, we discovered the existence of 2′‐*O*‐methyl‐5‐hydroxymethylcytidine (hm^5^Cm), a second methylated metabolite of m^5^C, in the RNA of higher organisms (Scheme 1).^12^ The formation of hm^5^Cm from m^5^C by stepwise oxidation via hm^5^C supports the dynamic nature and complexity of these cytosine modifications in RNA.^12^, ^13^ An efficient synthesis of oligonucleotides (ONs) containing these modifications is essential to elucidate the chemistry and function of RNA cytosine derivatives. For example, site‐specific incorporation of these cytosine modifications into RNA will enable the development of sequencing methods to decode the modification^14^ and also help identify the reader proteins so as to understand the cellular functions of these modifications.^8^

**Scheme 1:**
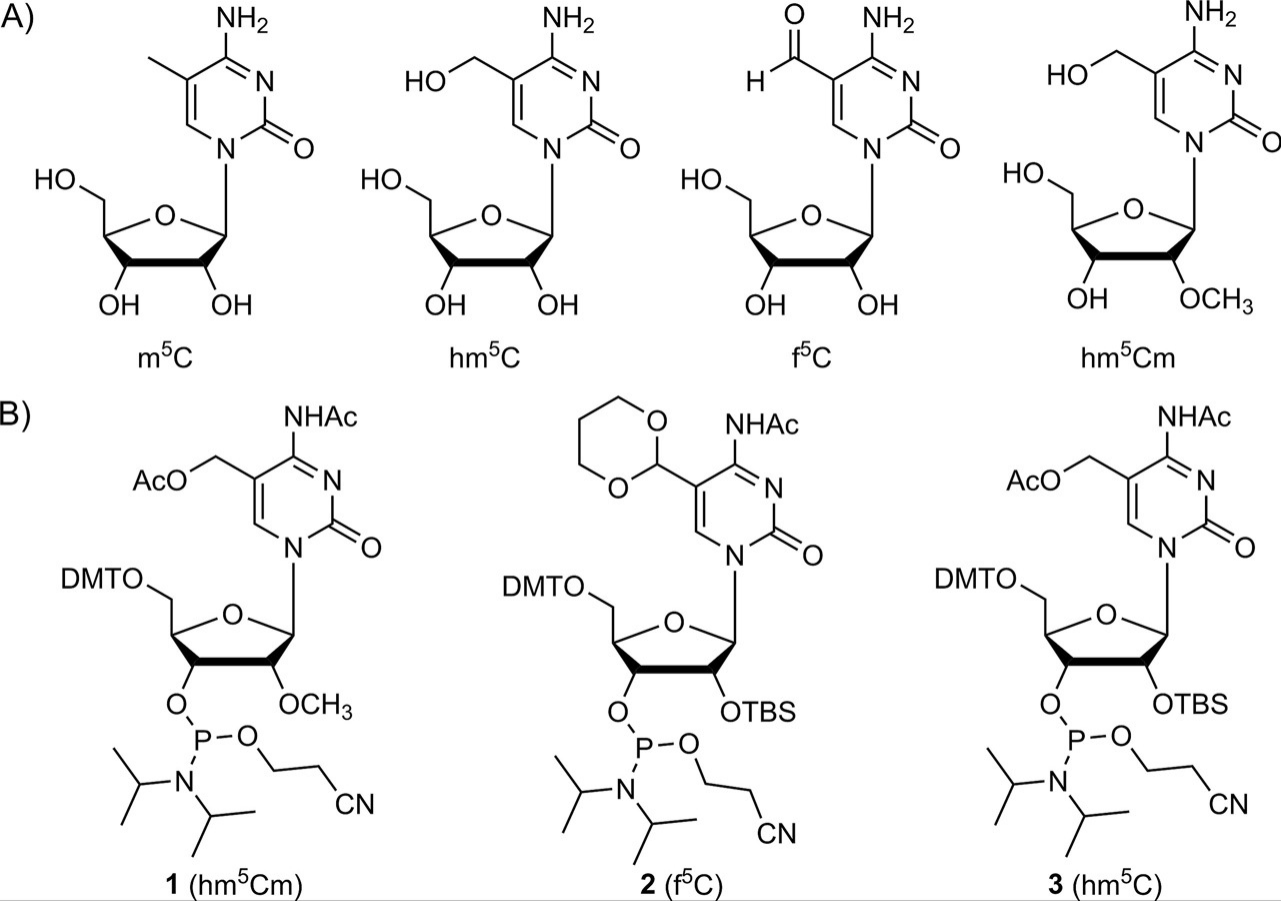
A) Chemical structure of major cytosine modifications known to exist in eukaryotic RNA. B) Corresponding phosphoramidite monomers synthesised in this work.

One phosphoramidite monomer used for the synthesis of m^5^C‐containing RNA ONs is commercially available, and syntheses of f^5^C and hm^5^C monomers have been reported in the literature.^10^, ^15^ We set out to develop a synthesis of a hm^5^Cm monomer that would be compatible with solid‐phase RNA ONs synthesis, which has not yet been described. In addition, the reported monomer for the synthesis of f^5^C‐containing RNA ONs requires a multistep synthesis with 2′‐bis(2‐acetoxyeth‐ oxy)methyl (ACE) orthoester and 5′‐*O*‐benzhydroxy‐bis(trimethylsiloxy)silyl (BZH) protection.^10^ One drawback of this monomer is the presence of a free formyl group that is susceptible to the oxidation and nucleophilic attack encountered during ON assembly and post‐synthetic resin cleavage, respectively.^10^, ^16^ Consequently, multiple incorporations by using the existing f^5^C phosphoramidite are difficult, especially in the synthesis of longer oligomers or in combination with other cytosine modifications such as hm^5^C and hm^5^Cm containing a nucleophilic moiety. To overcome these practical limitations, we designed an alternative f^5^C monomer in which the reactive formyl group is appropriately masked. Riml and Micura reported the synthesis and incorporation of a hm^5^C monomer into RNA ONs; their route provides this monomer in a 3 % overall yield in eight steps from 5‐hydroxymethyluridine (hm^5^U),^15^ which itself is obtained in a three‐step synthesis from uridine, and this brings the total number of steps to 11.^17^ We therefore, at the same time, considered the development of a faster and more efficient route to this monomer. The presence of the 2′‐hydroxy group makes the synthesis and incorporation of functionally modified ribonucleoside phosphoramidites particularly challenging compared to that of the analogous DNA phosphoramidite.^10^, ^15^, ^18^ Moreover, an orthogonal protection strategy developed for the synthesis of DNA ONs does not necessarily work for RNA due to its inherently more labile nature.18a Herein, we describe the development of phosphoramidite building blocks of hm^5^Cm, f^5^C and hm^5^C (Scheme 1) and demonstrate the incorporation of all these cytosine modifications into RNA ONs at several positions and in several combinations by solid‐phase RNA synthesis.

To synthesise hm^5^Cm phosphoramidite **1**, we decided to protect the 5‐hydroxymethyl with an acetyl group, which is compatible with the incorporation of hm^5^C into RNA ONs.^15^ Starting with commercially available 2′‐*O*‐methyl‐5‐methyluridine (**4**), we protected the 5′‐hydroxy group with a 4,4′‐dimethoxytrityl (DMT) and the 3′‐hydroxy group with a *tert*‐butyldimethylsilyl (TBS) group (Scheme 2).^19^ The DMT‐ and TBS‐protected uridine, **6**, was then subjected to azobisisobutyronitrile (AIBN)‐catalysed bromination of the 5‐methyl group by using *N*‐bromosuccinimide (NBS).^20^ The crude bromo derivative was treated with potassium acetate to yield the fully protected uridine analogue **7** in moderate yield.^19^ The reaction of **7** with 2,4,6‐triisopropylbenzenesulfonyl (TPS) chloride resulted in regioselectively O^4^‐trisylated compound that was readily converted into cytidine analogue **8** upon ammonolysis. The exocyclic amino group was acetylated with acetic anhydride in pyridine to provide nucleoside **9**. Cleavage of the 3′‐*O*‐TBS group with TBAF and acetic acid in THF gave precursor **10**. Finally, phosphitylation of the 3′‐hydroxy group with 2‐cyanoethyl‐*N*,*N*‐diisopropylchloro‐phosphoramidite (CEP‐Cl) in the presence of *N*,*N*‐diisopropylethylamine afforded phosphoramidite **1** in a 6.7 % overall yield in seven steps from **4**.^19^

**Scheme 2:**
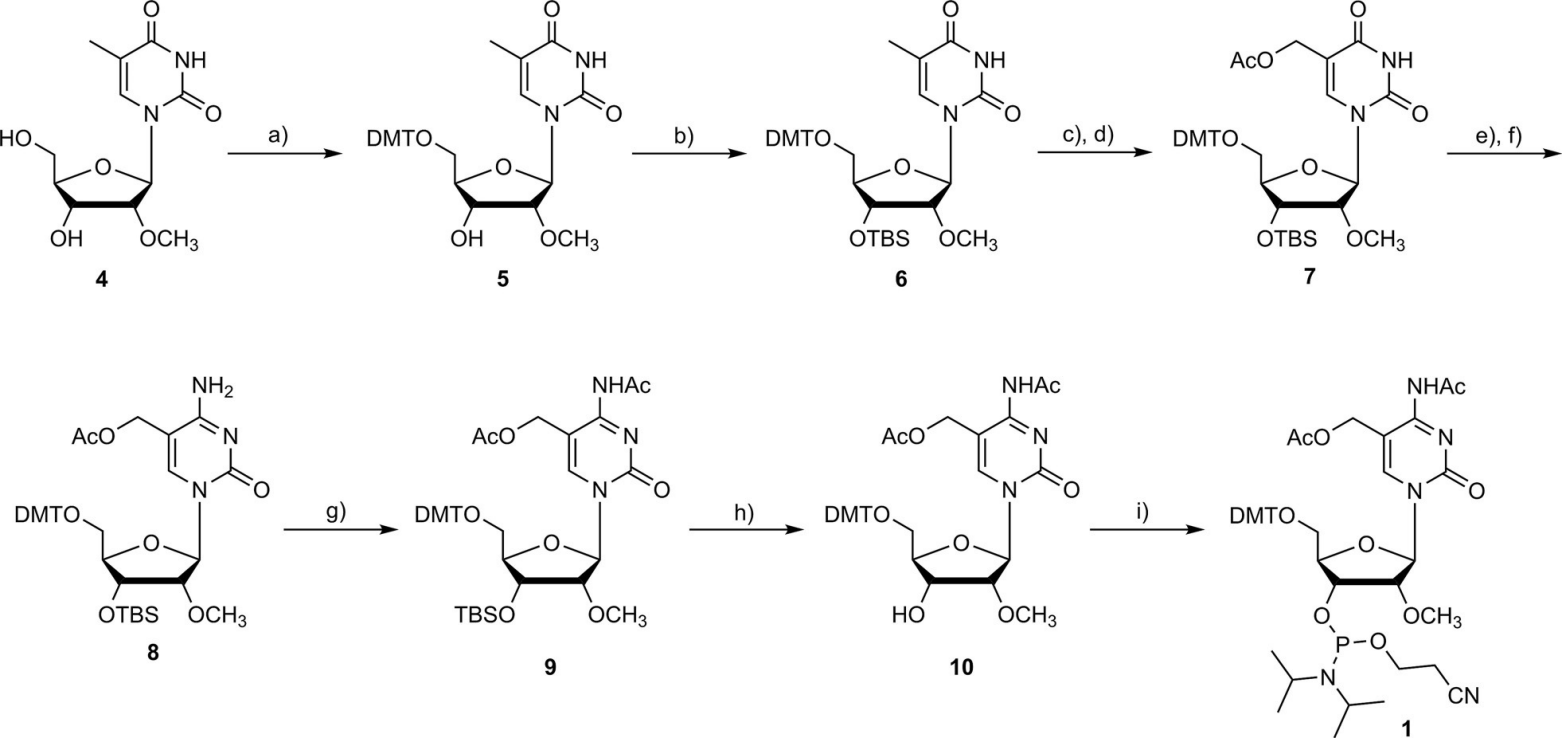
Synthesis of hm^5^Cm phosphoramidite **1**.^19^ a) DMT‐Cl, 4‐dimethylaminopyridine (DMAP), pyridine, RT (90 %); b) TBS‐Cl, imidazole, DMF, RT (83 %); c) NBS, AIBN, benzene, 80 °C; d) CH_3_CO_2_K, DMF, 50 °C (42 %); e) TPS‐Cl, Et_3_N, DMAP, CH_2_Cl_2_, RT; f) aq. NH_3_, THF, RT (42 %); g) Ac_2_O, pyridine, 0 °C–RT (94 %); h) 1 m TBAF, 0.5 m AcOH, THF, RT (75 %); i) CEP‐Cl, *i*Pr_2_NEt, CH_2_Cl_2_, RT (72 %).

In DNA synthesis, acetal chemistry has been successfully used to protect the formyl group.^21^ To explore the analogous protection of f^5^C for RNA, we synthesised phosphoramidite building block **2** (Scheme 3).^19^ Starting from 5‐methyluridine (**11**), we protected the 3′‐ and 5′‐hydroxy groups with di‐*tert*‐butylsilylene and subsequently protected the 2′‐hydroxy group with TBS.^22^ Nucleoside **12** was selectively brominated at the C5‐methyl group, then acylation yielded uridine‐derivative **13** in good yield. Hydrolysis of **13** provided 5‐hydroxymethyl derivative **14**, which was then converted into aldehyde **15** by Dess–Martin periodinane (DMP) oxidation.^23^ Next, the formyl group was protected as a 1,3‐dioxane by using propane‐1,3‐diol in the presence of TiCl_4_, according to the procedure described for synthesising an analogous 2′‐deoxy phosphoramidite.^21^ Fully protected uridine analogue **16** was then treated with TPS‐Cl, followed by ammonolysis to give cytidine analogue **17**. The exocyclic amino group was acetylated with acetic anhydride in pyridine to provide **18**.

**Scheme 3:**
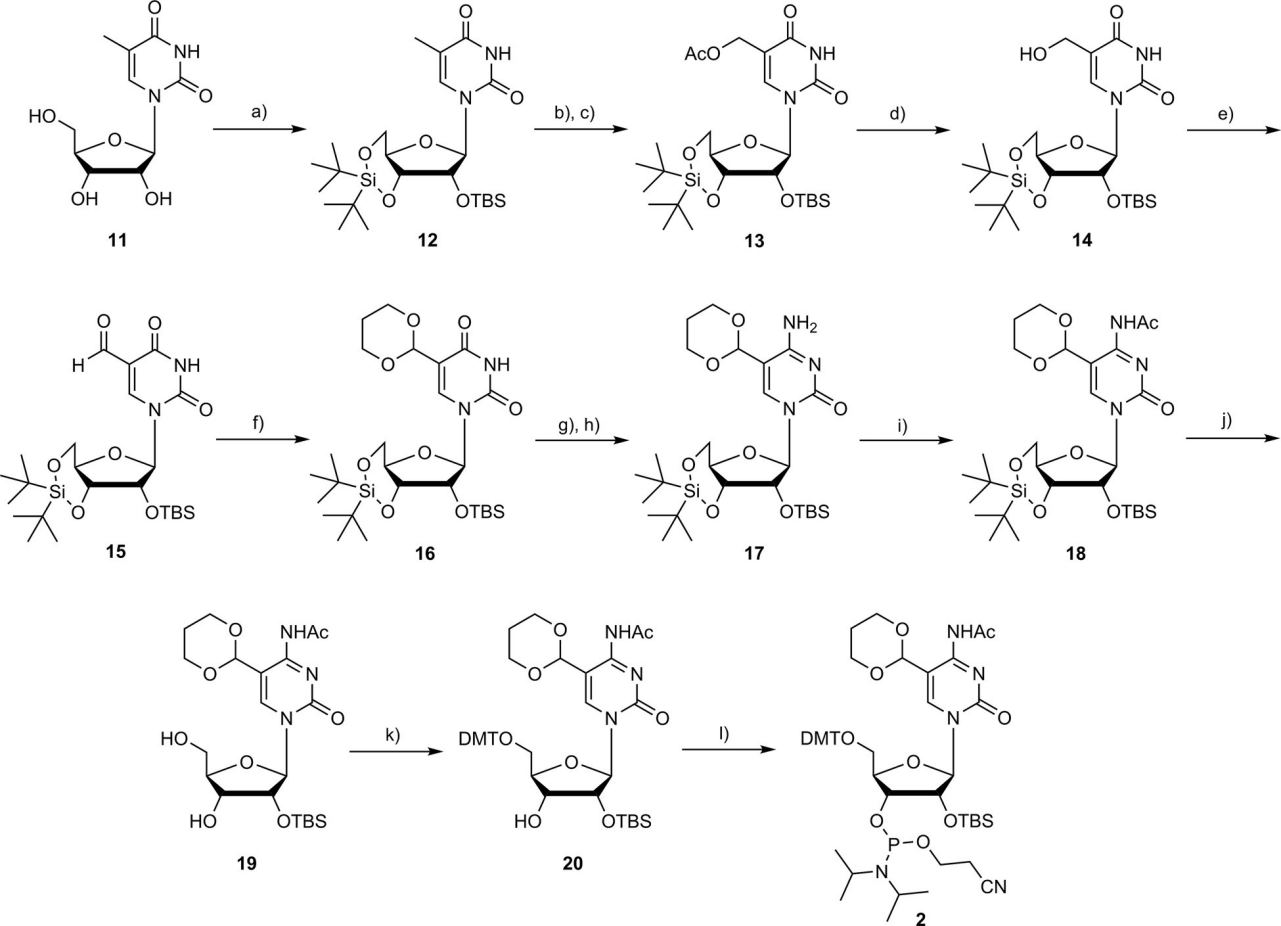
Synthesis of f^5^C phosphoramidite **2**.^19^ a) (*t*Bu)_2_Si(OTf)_2_, TBS‐Cl, imidazole, DMF, RT (91 %); b) NBS, AIBN, benzene, 80 °C; c) CH_3_CO_2_K, DMF, 50 °C (64 %); d) K_2_CO_3_, H_2_O, THF, RT (92 %); e) DMP, CH_2_Cl_2_, 0 °C→RT (77 %); f) propane‐1,3‐diol, triethyl orthoformate, TiCl_4_, CH_2_Cl_2_, 0 °C (88 %); g) TPS‐Cl, Et_3_N, DMAP, CH_2_Cl_2_, RT; h) aq NH_3_, THF, RT (67 %); i) Ac_2_O, pyridine, 0 °C→RT (95 %); j) HF**⋅**pyridine, CH_2_Cl_2_, 0 °C (95 %); k) DMT‐Cl, DMAP, pyridine, RT (63 %); l) CEP‐Cl, *i*Pr_2_NEt, CH_2_Cl_2_, RT (76 %).

The 3′, 5′‐*O*‐di‐*tert*‐butylsilylene group was selectively removed by treating **18** with HF**⋅**pyridine. Finally, 5′‐OH DMT protection and subsequent phosphitylation yielded target monomer **2**. Starting from 5‐methyluridine and following this ten‐step route, we obtained monomer **2** in a good 10.5 % overall yield (Scheme 3).^19^ Compared to the previously reported synthesis for an f^5^C phosphoramidite, we have significantly improved the overall yield and, more importantly, we have now deployed a formyl protective group.^10^ We anticipated that this fully protected monomer would allow us to incorporate f^5^C at multiple positions in longer ONs and also in the combination with the other cytosine modifications m^5^C, hm^5^C and hm^5^Cm.

Next, we synthesised the hm^5^C phosphoramidite **3** (Scheme 4). During the synthesis of the previous two monomers, we noted that the methyl group of appropriately protected 5‐methyluridine could be functionalised efficiently. Hence, we started our synthesis by protecting the 5′‐hydroxy group of 5‐methyluridine (**11**) with DMT, to give **21**, and protecting the 2′‐ and 3′‐hydroxy groups with TBS. Conversion of the 5‐methyl group of **22** into the 5‐acetyloxymethyl group was the key step in this synthesis, and we achieved it by bromination and subsequent acylation.^19^ From here onwards we followed the route reported by Riml et al. and obtained monomer **3** in eight steps with 6.4 % overall yield (Scheme 4).^15^, ^19^ During the preparation of this manuscript, the Micura group improved their initial synthesis and reported an alternative method, starting from cytidine, to obtained this monomer in eight steps with 9.2 % overall yield.^24^

**Scheme 4:**
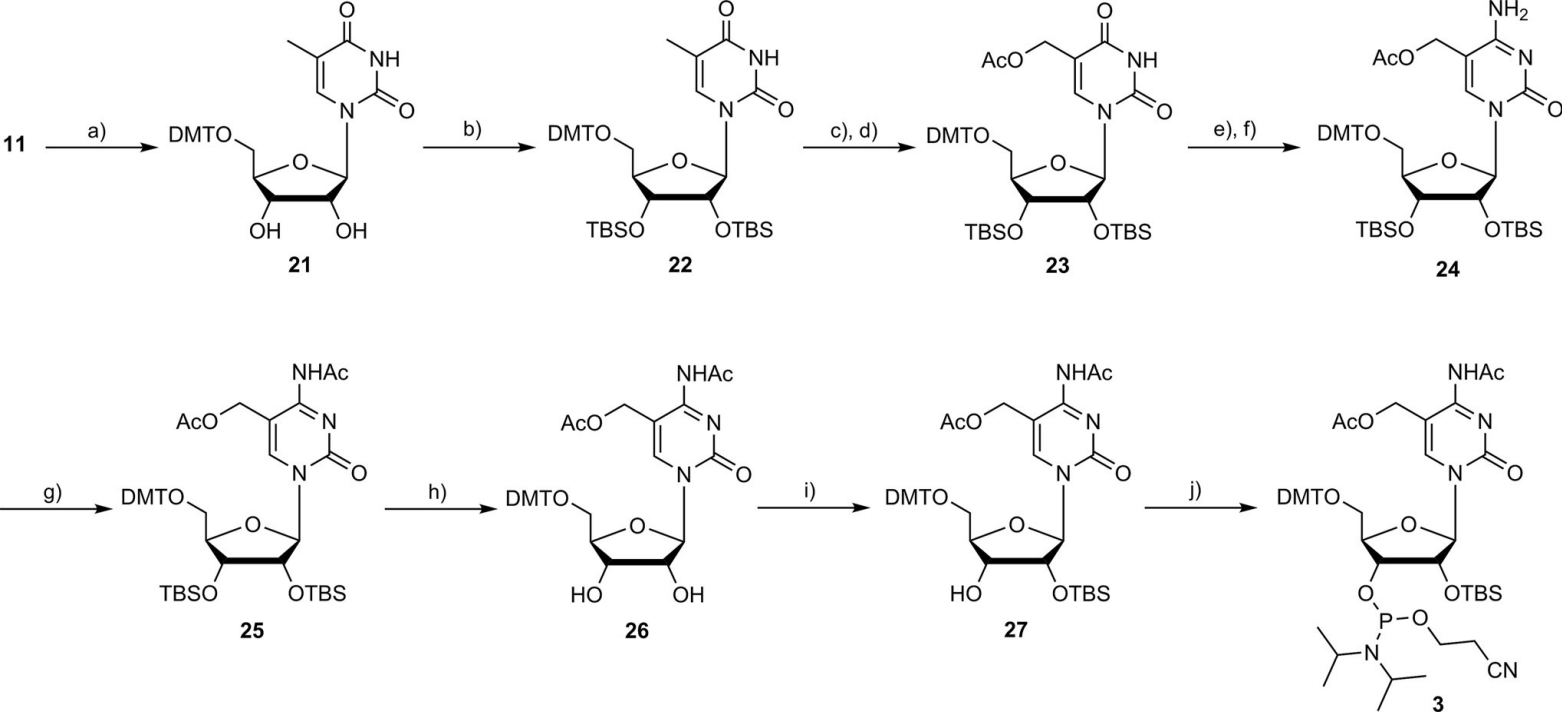
Synthesis of hm^5^C phosphoramidite **3**.^19^ a) DMT‐Cl, DMAP, pyridine, RT, 83 %); b) TBS‐Cl, imidazole, DMF, RT (87 %); c) NBS, AIBN, benzene, 80 °C; d) CH_3_CO_2_K, DMF, 50 °C (65 %); e) TPS‐Cl, Et_3_N, DMAP, CH_2_Cl_2_, RT; f) aq NH_3_, THF, RT (43 %); g) Ac_2_O, pyridine, 0 °C→RT (78 %); h) 1 m TBAF, 0.5 m AcOH, THF, RT (95 %); i) AgNO_3_, TBS‐Cl, pyridine, THF, RT (56 %); j) CEP‐Cl, *i*Pr_2_NEt, CH_2_Cl_2_, RT (76 %).

To evaluate the utility of our building blocks for obtaining ONs containing various modifications we synthesised a small series of RNA ONs (Figure 1 A). To further confirm the robustness of these monomers, we synthesised RNA 1–RNA 3, which each contain a single modification at three different positions, and RNA 4, which contains all four cytosine modifications. To ascertain the compatibility of these monomers in the presence of a biochemical tag, we also performed the RNA ON synthesis on CPG solid support tethered to triethylene glycol (TEG)‐biotin. Modified phosphoramidites were incorporated into RNA ONs according to a standard solid‐phase RNA ON synthesis protocol.^25^ Modified phosphoramidite substrates **1**–**3** were incorporated with a coupling time of 10 min, with 80–90 % coupling efficiency, based on a trityl release assay. Oligonucleotides were cleaved from the solid support with 20 % ethanol in NH_4_OH. 2′‐*O*‐TBS deprotection was performed with a 1:1 mixture of anhydrous DMSO and triethylamine trihydrofluoride. RNA 1 and RNA 3 were purified by HPLC at this stage, whereas RNA 2 and 4 were each subjected to deprotection of the acetal moiety to reveal the formyl group.^19^ Conditions previously used for the acetal deprotection of fdC in DNA led to only partial deprotection of the acetal group for RNA 2 and RNA 4, along with precipitation of RNA. After screening several deprotection conditions, we found that treating RNA ON with 20 % aqueous acetic acid results in complete and clean removal of the acetal group without cleavage of the RNA (Figure S1 in the Supporting Information). The purity and integrity of all modified RNA ONs were confirmed by LC‐MS analysis (Figures 1, S1 and S2).^19^

**Figure 1:**
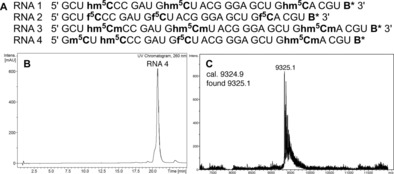
A) Sequences of the modified RNA ONs synthesised in this study. RNAs 1–3 contain hm^5^C, f^5^C or hm^5^Cm, respectively, at three different positions; RNA 4 contains all four cytosine modifications. The 3′‐end of each RNA ON is tagged with TEG‐biotin (B*). B) Representative LC trace of RNA 4. C) Corresponding ESI‐MS spectrum of RNA 4.^19^

To further demonstrate, beyond mass spectroscopy, that the modified ribonucleosides remained intact upon RNA synthesis and deprotection, we performed a post‐synthetic chemical functionalisation of the RNA. Firstly, we subjected the RNA 2, which contains a reactive formyl group, to nucleophile addition with ethoxyamine hydrochloride at pH 5.0 in the presence of anisidine.^26^ All three f^5^C nucleotides in RNA 2 undergo facile addition of ethoxyamine, followed by elimination of water to form a stable imine derivative, which we confirmed by LC‐MS analysis (Figure 2).^19^, ^26^ Next, we performed the same reaction with RNAs 1 and 3, and observed that both the RNA ONs remain unaltered. This experiment supports the functional integrity of these modifications in RNA.

**Figure 2:**
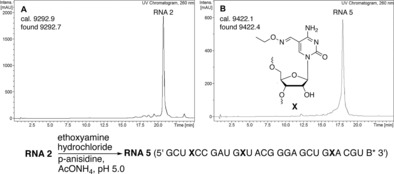
LC‐MS chromatogram of RNA 2 A) before and B) after post‐synthetic chemical reaction with ethoxyamine hydrochloride at pH 5.0.^26^ The sequence of RNA 5 is given at the bottom, and the structure of the modified nucleoside (**X**) formed upon selective condensation between f^5^C and ethoxyamine is shown in (B).^19^

In summary, we have demonstrated the efficient syntheses of hm^5^Cm, hm^5^C, and f^5^C building blocks and the synthesis of RNA ONs containing these modifications at multiple positions in excellent yield and purity. The availability of these monomers and the ability to prepare RNA oligomers with any combination, in desired positions, will be important for studying the function(s) of cytosine modifications in biology.

## Conflict of interest

*S.B. is a founder and shareholder of Cambridge Epigenetix Ltd*.

## Supporting information

As a service to our authors and readers, this journal provides supporting information supplied by the authors. Such materials are peer reviewed and may be re‐organized for online delivery, but are not copy‐edited or typeset. Technical support issues arising from supporting information (other than missing files) should be addressed to the authors.

Supplementary
