# Supplementary material for: Synthesis and Multiple Incorporations of 2′‐O‐Methyl‐5‐hydroxymethylcytidine, 5‐Hydroxymethylcytidine and 5‐Formylcytidine Monomers into RNA Oligonucleotides
Source: Chembiochem. 2017 Oct 9;18(22):2236–41. doi: 10.1002/cbic.201700492 (PMC5779611; doi:10.1002/cbic.201700492)

CHEMBIOCHEM

Supporting Information

Synthesis and Multiple Incorporations of 2′-*O*-Methyl-5-hydroxymethylcytidine, 5-Hydroxymethylcytidine and 5-Formylcytidine Monomers into RNA Oligonucleotides

Arun A. Tanpure[a] and Shankar Balasubramanian*[a, b, c]

cbic_201700492_sm_miscellaneous_information.pdf

Contents	Page
**1. Materials and Instrumentation**	S2
**2. Synthesis**	S2
**Scheme S1**. Synthesis of hm^5Cm phosphoramidite **1** and related compounds	S2
**Scheme S2**. Synthesis of f^5C phosphoramidite **2** and related compounds	S5
**Scheme S3**. Synthesis of hm^5C phosphoramidite **3** and related compounds	S9
**3. Solid-phase synthesis of RNA ONs**	S11
**4. LC-MS analysis of RNA ONs**	S12
**Figure S1**. LC trace and ESI-MS spectrum of RNA ONs after HPLC purification	S12
**Figure S2**. HPLC chromatograms of RNA ONs **2** and **4** after acetal deprotection	S13
**5. Postsynthetic chemical functionalisation of RNA ONs**	S13
**6. References**	S13
**7. NMR spectra**	S14

1. Materials and Instrumentation

All solvents and reagents were purchased from either Sigma Aldrich or Fisher Scientific and used as received. Unless mentioned all reactions were performed at ambient temperature and under nitrogen atmosphere. TLCs were performed on ALUGRAM SIL G/UV254 (Macherey-Nagel) pre-coated TLC sheets. Flash chromatographic purifications were carried out using CombiFlash *Rf* (Teledyne Isco) with RediSep*Rf* column. HRMS spectra were recorded on Micromass® Q-Tof (ESI) spectrometer (Waters). NMR spectra were recorded on a Bruker DPX-400 instrument and are referenced to the residual solvent peak. Chemical shifts are quoted in parts per million (ppm) using the following abbreviations: s, singlet; d, doublet; dd, doublet of double; t, triplet; m, multiplet; br, broad. The coupling constants (J) are measured in Hertz. Modified RNA oligonucleotides (ONs) were synthesized on an Applied Biosystems RNA/DNA synthesizer (ABI-394). HPLC analyses were performed using Agilent Technologies 1260 Infinity. Absorption spectra were recorded on a Cary 100 UV-Visible spectrophotometer from Agilent Technologies. LC-ESIMS spectra were recorded on an Amazon ESI-MS (Bruker) connected to Ultimate 3000 LC (Dionnex).

2. Synthesis

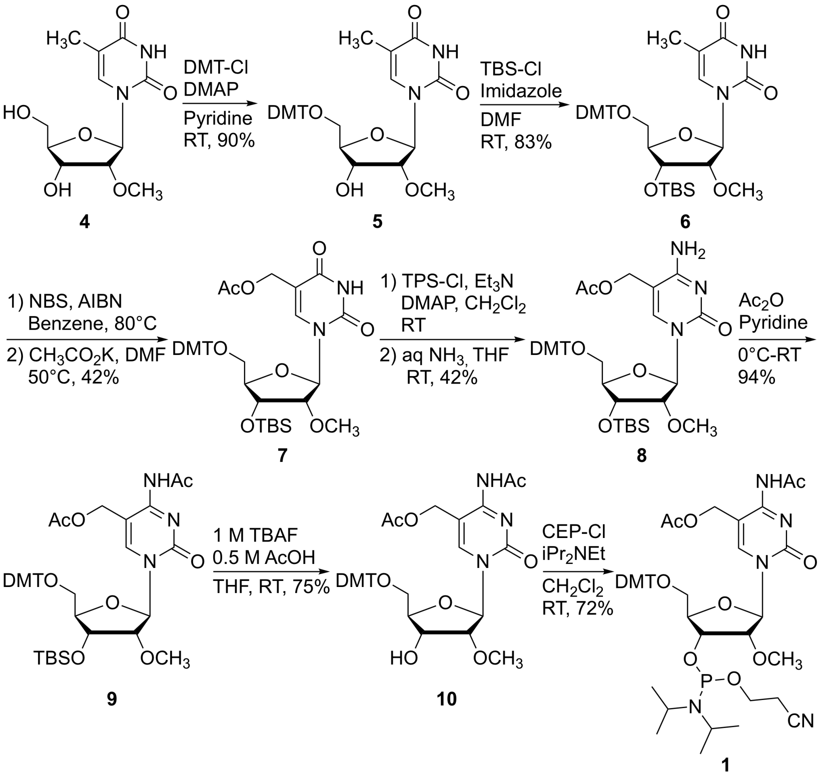

Scheme S1. Synthesis of hm^{5}Cm phosphoramidite **1** and related compounds.

5'-*O*-(4,4'- dimethoxytrityl)-2'-*O*-methyl-5-methyluridine (5)[S1]**:** A solution of 2'-*O*-methyl-5-methyluridine **4** (2.00 gm, 7.35 mmol), DMT-Cl (3.24 gm, 9.55 mmol) and DMAP (90 mg, 0.735 mmol) in anhydrous pyridine (15 mL) was stirred at room temperature under nitrogen atmosphere for overnight. Pyridine was evaporated under reduced pressure. Crude mixture was then diluted with dichloromethane (50 mL) and washed with 5% sodium bicarbonate solution followed by brine (30 mL each). Organic extract was dried over sodium

sulphate, evaporated and purified by flash column chromatography (0−5 % methanol in dichloromethane containing 0.5% triethylamine) to afford the product **5** as a white solid (3.80 gm, 90%).

5'-*O*-(4,4'-dimethoxytrityl)-3'-*O*-*tert*-butyldimethylsilyl-2'-*O*-methyl-5-methyluridine (6): To a solution of compound **5** (3.7 gm, 6.44 mmol) in dry DMF (37 mL), Imidazole (1.14 gm, 16.74 mmol) and TBS-Cl (1.26 gm, 8.37 mmol) were added and the reaction was allowed to proceed for overnight at room temperature. All volatiles were evaporated under reduced pressure and residue was partitioned between ethyl acetate (100 mL) and water (70 mL). The organic layer was separated, washed with brine (70 mL), dried over sodium sulfate, filtered and evaporated. Crude product was purified by flash column chromatography (10−25% ethyl acetate in hexane) to yield the compound **6** as a white foam (3.68 gm, 83%). TLC (Hexane:EtOAc = 70:30); R_f = 0.5.; **^{1}H-NMR (400 MHz, DMSO-d$_6$):** δ (ppm) 11.41 (s, 1H), 7.60 (s, 1H), 7.40−7.24 (m, 9H), 6.90 (dd, *J* = 9.2 Hz, 2.4 Hz, 4H), 5.80 (d, *J* = 3.6 Hz, 1H), 4.36 (t, *J* = 5.8 Hz, 1H), 3.93−3.88 (m, 2H), 3.73 (s, 6H), 3.38 (s, 3H), 3.34 (d, *J* = 2.4 Hz, 1H), 3.14 (dd, *J* = 10.6 Hz, 4.2 Hz, 1H), 1.44 (s, 3H), 0.76 (s, 9H), 0.03 (s, 3H), 0.04 (s, 3H).; **^{13}C-NMR (100 MHz, DMSO-d$_6$):** δ (ppm) 163.8, 158.3, 150.3, 144.6, 135.8, 135.3, 135.2, 129.9, 128.0, 127.8, 127.0, 113.3, 109.5, 87.6, 86.0, 82.6, 82.1, 69.8, 57.8, 55.2, 25.6, 17.8, 11.9, -4.6, -5.1.; **HRMS (ESI+):** Calculated for $C_{38}H_{49}N_2O_8Si$ $[M+H]^+$: 689.3258, found: 689.3259.

5'-*O*-(4,4'-dimethoxytrityl)-3'-*O*-*tert*-butyldimethylsilyl-2'-*O*-methyl-5-acetyloxy-methyl-uridine (7): To a solution of **6** (3.6 gm, 5.23 mmol) in dry benzene (54 mL), *N*-Bromosuccinimide (1.12 gm, 6.27 mmol) and AIBN (103 mg, 0.63 mmol) were added at 60 °C. Reaction mixture was refluxed for 40 min before cooled to room temperature. Solvent was evaporated under reduced pressure. Crude bromo-derivative was dissolved in dry DMF (18 mL) and then potassium acetate (1.03 gm, 10.46 mmol) was added, resulting slurry was stirred at 50 °C for 30 min. Reaction mixture was diluted with ethyl acetate (50 mL) and washed with water followed by brine (40 mL each), dried over anhydrous sodium sulfate, filtered and evaporated. Flash column chromatography purification (10−30% ethyl acetate in hexane) afforded compound **7** (1.64 gm, 42%) as a white solid. TLC (Hexane:EtOAc = 70:30); R_f = 0.45.; **^{1}H-NMR (400 MHz, DMSO-d$_6$):** δ (ppm) 11.57 (s, 1H), 7.85 (s, 1H), 7.38 (d, *J* = 7.2 Hz, 2H), 7.32−7.21 (m, 7H), 6.87 (d, *J* = 8.0 Hz, 4H), 5.79 (d, *J* = 2.8 Hz, 1H), 4.39 (d, *J* = 11.6 Hz, 1H), 4.30−4.25 (m, 2H), 3.95−3.89 (m, 2H), 3.72 (s, 6H), 3.39 (s, 3H), 3.31 (d, *J* = 2.4 Hz, 1H), 3.19 (dd, *J* = 10.8 Hz, 4.8 Hz, 1H), 1.85 (s, 3H), 0.75 (s, 9H), 0.02 (s, 3H), 0.06 (s, 3H).; **^{13}C-NMR (100 MHz, DMSO-d$_6$):** δ (ppm) 170.0, 162.5, 158.2, 150.0, 144.4, 140.9, 135.3, 129.7, 127.8, 126.8, 113.2, 108.1, 88.2, 85.8, 82.3, 82.0, 69.7, 62.3, 58.7, 57.8, 55.0, 25.5, 20.5, 17.6, -4.7, -5.2.; **HRMS (ESI+):** Calculated for $C_{40}H_{51}N_2O_{10}Si$ $[M+H]^+$: 747.3313, found: 747.3308.

5-Acetyloxymethyl-5'-*O*-(4,4'-dimethoxytrityl)-3'-*O*-*tert*-butyldimethylsilyl-2'-*O*-methylcytidine (8): Compound **7** (1.60 gm, 2.14 mmol) and DMAP (26 mg, 0.21 mmol) were dissolved in dry dichloromethane (16 mL) and triethylamine (3.0 mL) solution. Trisyl-chloride (972 mg, 3.21 mmol) was added in one portion and stirring was continued for 2 h at room temperature. Reaction mixture was diluted with dichloromethane (20 mL), washed with saturated sodium bicarbonate solution and brine (40 mL each), dried over anhydrous sodium sulfate, filtered and evaporated. Next, the crude product was dissolved in a mixture of THF (25 mL) and 32% aqueous ammonia (40 mL) and stirred for 2 h at room temperature. Final

product was extracted in ethyl acetate (50 mL) and washed with saturated sodium bicarbonate and brine (50 mL each), dried over anhydrous sodium sulfate, filtered and evaporated. Flash column chromatography (2–6 % methanol in dichloromethane) provided compound **8** as a white solid (0.67 gm, 42%). TLC (CH_2Cl_2:MeOH = 94:6); R_f = 0.4.; **^{1}H-NMR (400 MHz, DMSO-d$_6$):** δ (ppm) 7.80 (s, 1H), 7.54 (br, 1H), 7.39 (d, *J* = 7.2 Hz, 2H), 7.31–7.22 (m, 7H), 7.02 (br, 1H), 6.88 (dd, *J* = 9.5 Hz, 1.5 Hz, 4H), 5.84 (d, *J* = 2.5 Hz, 1H), 4.39 (d, *J* = 10.0 Hz, 1H), 4.34 (d, *J* = 10.0 Hz, 1H), 4.24 (dd, *J* = 5.8 Hz, 4.0 Hz, 1H), 3.94–3.92 (m, 1H), 3.73 (s, 6H), 3.41 (s, 3H), 3.30 (d, *J* = 2.0 Hz, 1H), 3.21–3.18 (m, 2H), 1.90 (s, 3H), 0.74 (s, 9H), 0.00 (s, 3H), -0.08 (s, 3H).; **^{13}C-NMR (100 MHz, DMSO-d$_6$):** δ (ppm) 170.1, 164.4, 158.2, 154.4, 144.4, 142.4, 135.3, 135.2, 129.7, 127.8, 126.8, 113.2, 100.4, 88.4, 85.8, 82.6, 81.9, 69.8, 62.5, 60.4, 57.7, 55.0, 54.9, 25.5, 20.7, 17.6, -4.7, -5.3.; **HRMS (ESI+):** Calculated for $C_{40}H_{51}N_3O_9SiK$ $[M+K]^+$: 784.3032, found: 784.3028.

***N^4*-Acetyl-5-acetyloxymethyl-5'-*O*-(4,4'-dimethoxytrityl)-3'-*O*-*tert*-butyldimethylsilyl-2'-O-methylcytidine (9)**: Compound **8** (650 mg, 0.87 mmol) was dissolved in dry pyridine (10 mL) and cooled to 0 °C. Acetic anhydride (205 µL, 2.18 mmol) was slowly added to the above solution, after addition cooling was removed and reaction was stirred at room temperature for 4 h before quenched by methanol. Solvents were evaporated completely under reduced pressure and crude product was purified by flash column chromatography (1–5 % methanol in dichloromethane) to afforded compound **9** as a white solid (0.65 gm, 94%). TLC (CH_2Cl_2:MeOH = 95:5); R_f = 0.5.; **^{1}H-NMR (400 MHz, DMSO-d$_6$):** δ (ppm) 10.10 (s, 1H), 8.18 (s, 1H), 7.39 (d, *J* = 7.0 Hz, 2H), 7.31 (d, *J* = 7 Hz, 2H), 7.27–7.24 (m, 5H), 6.88 (dd, *J* = 8.4 Hz, 1.6 Hz, 4H), 5.81 (br, 1H), 4.60 (d, *J* = 10.4 Hz, 1H), 4.34 (d, *J* = 10.8 Hz, 1H), 4.27 (dd, *J* = 6.6 Hz, 4.2 Hz, 1H), 4.02–4.00 (m, 1H), 3.84 (d, *J* = 3.6 Hz, 1H), 3.73 (s, 6H), 3.47 (s, 3H), 3.40 (d, *J* = 7.6 Hz, 1H), 3.22 (dd, *J* = 8.8 Hz, 3.6 Hz, 1H), 2.25 (s, 3H), 1.82 (s, 3H), 0.72 (s, 9H), 0.01 (s, 3H), -0.10 (s, 3H).; **^{13}C-NMR (100 MHz, DMSO-d$_6$):** δ (ppm) 170.6, 169.9, 161.3, 158.2, 153.5, 149.6, 145.6, 144.2, 135.2, 135.1, 129.7, 127.8, 126.9, 113.2, 104.6, 89.6, 85.8, 82.5, 82.1, 69.2, 61.7, 60.2, 57.9, 55.0, 54.9, 25.5, 24.9, 20.5, 17.6, -4.7, -5.4.; **HRMS (ESI+):** Calculated for $C_{42}H_{53}N_3O_{10}SiK$ $[M+K]^+$: 826.3137, found: 826.3139.

***N^4*-Acetyl-5-acetyloxymethyl-5'-*O*-(4,4'-dimethoxytrityl)-2'-*O*-methylcytidine (10):** Compound **9** (620 mg, 0.79 mmol) was dissolved in a solution of 1 M TBAF (3 mL) containing 0.5 M AcOH in THF and stirred at 35 °C for 2 h. Solvents were evaporated and the residue was dissolved in dichloromethane (20 mL), washed with water followed by brine (15 mL each). The organic layer was dried over anhydrous sodium sulfate, filtered and evaporated. Crude product was purified by flash column chromatography (2–5 % methanol in dichloromethane) to afforded compound **10** as a white solid (400 mg, 75%). TLC (CH_2Cl_2:MeOH = 95:5); R_f = 0.4.; **^{1}H-NMR (400 MHz, DMSO-d$_6$):** δ (ppm) 10.08 (s, 1H), 8.08 (s, 1H), 7.40 (d, *J* = 5.6 Hz, 2H), 7.32–7.21 (m, 7H), 6.88 (d, *J* = 6.8 Hz, 4H), 5.83 (d, *J* = 2.0 Hz, 1H), 5.20 (d, *J* = 6.0 Hz, 1H), 4.60 (d, *J* = 10.4 Hz, 1H), 4.35 (d, *J* = 10.4 Hz, 1H), 4.18 (dd, *J* = 10.4 Hz, 6.0 Hz, 1H), 4.07–4.03 (m, 1H), 3.80 (d, *J* = 5.2 Hz, 1H), 3.74 (s, 6H), 3.50 (s, 3H), 3.34 (dd, *J* = 8.6 Hz, 4.2 Hz, 1H), 3.25 (d, *J* = 7.6 Hz, 1H), 2.25 (s, 3H), 1.87 (s, 3H).; **^{13}C-NMR (100 MHz, DMSO-d$_6$):** δ (ppm) 170.6, 169.9, 161.3, 158.1, 153.5, 145.4, 144.5, 135.5, 135.2, 129.7, 127.9, 127.7, 126.8, 113.2, 104.6, 88.9, 85.7, 83.0, 82.2, 68.3, 62.4, 60.1, 58.0, 55.0, 54.9, 24.9, 20.5.; **HRMS (ESI+):** Calculated for $C_{36}H_{39}N_3O_{10}K$ $[M+K]^+$: 712.2273, found: 712.2277.

N^4-Acetyl-5-acetyloxymethyl-5′-*O*-(4,4′-dimethoxytrityl)-2′-*O*-methylcytidine-3′-*O*-(2-cyanoethyl-*N,N*-diisopropylphosphoramidite) (1): To a solution of compound **10** (280 mg, 0.42 mmol) in anhydrous dichloromethane (3 mL) was added *N,N*-diisopropylethylamine (0.360 μL, 2.08 mmol) and stirred for 5 min. To this solution 2-cyanoethyl *N,N*-diisopropylchlorophosphoramidite (150 μL, 0.62 mmol) was slowly added and reaction mixture was stirred for 5 h. Solvent was evaporated to dryness and residue was dissolved in ethyl acetate (20 mL), which was washed with 5% sodium bicarbonate followed by brine (15 mL each). Organic extract was dried over anhydrous sodium sulphate, filtered and evaporated. Crude residue was purified by flash column chromatography (25−55% ethyl acetate in hexane containing 1% triethylamine) to afford the product **1** as a white solid (262 mg, 72 %). TLC (Hexane:EtOAc = 25:75 with few drops of triethylamine); R_f = 0.5.; **^{1}H-NMR (400 MHz, DMSO-d$_6$):** δ (ppm) 10.05 (s, 1H), 8.15 (s, 1H), 7.40 (d, *J* = 8.4 Hz, 2H), 7.32−7.24 (m, 7H), 6.88 (dd, *J* = 6.8 Hz, 2.4 Hz, 4H), 5.87 (d, *J* = 2.0 Hz, 1H), 4.60 (d, *J* = 10.4 Hz, 1H), 4.39 (d, *J* = 10.4 Hz, 1H), 4.31 (dd, *J* = 6.8 Hz, 2.0 Hz, 2H), 4.17 (br, 1H), 4.02−3.96 (m, 1H), 3.73 (s, 6H), 3.62−3.55 (m, 1H), 3.49 (s, 3H), 3.38 (d, *J* = 7.2 Hz, 1H), 2.76 (d, *J* = 7.6 Hz, 1H), 2.25 (s, 3H), 1.86 (s, 3H), 1.08 (d, *J* = 5.6 Hz, 12H), 0.92 (d, *J* = 5.6 Hz, 4H).; **^{31}P-NMR (162 MHz, DMSO-d$_6$):** δ (ppm) 148.80, 148.54.; **HRMS (ESI+):** Calculated for $C_{45}H_{57}N_5O_{11}P$ $[M+H]^+$: 874.3792, found: 874.3799.

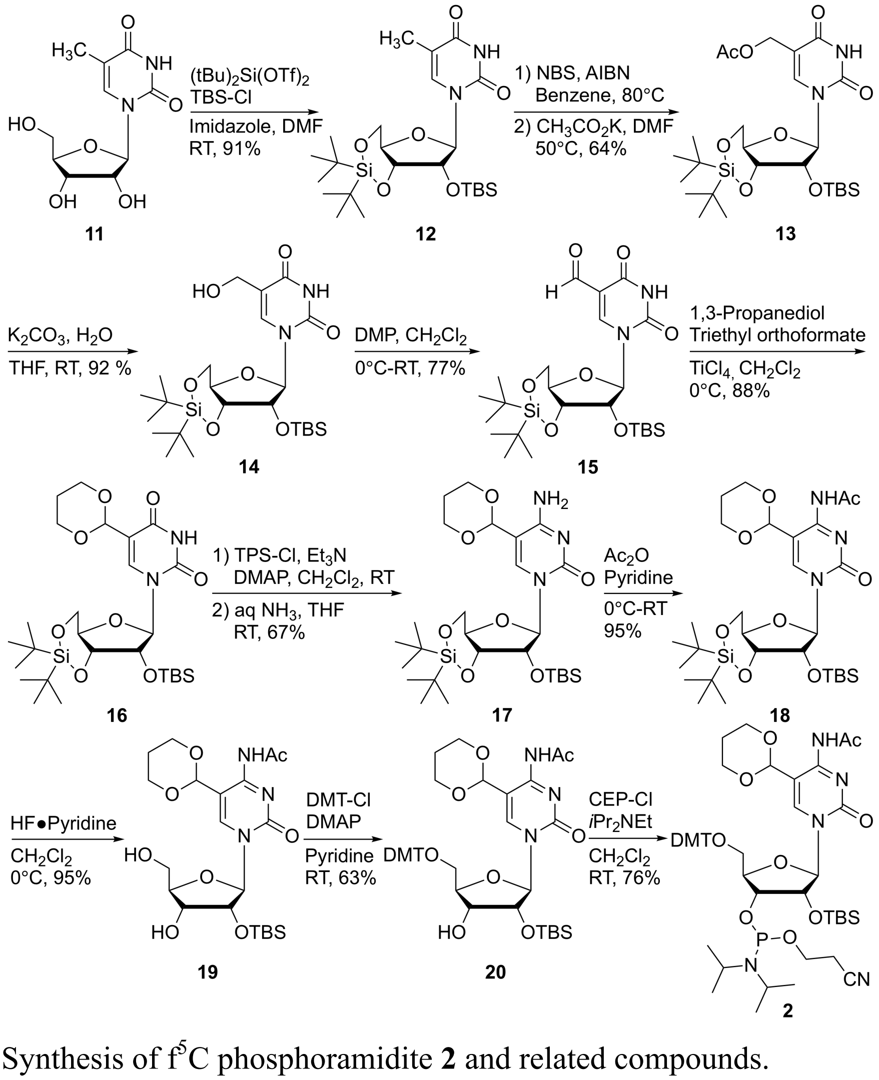

Scheme S2. Synthesis of f^{5}C phosphoramidite **2** and related compounds.

3',5'-*O*-(di-*tert*-butylsilylene)-2'-*O*-(*tert*-butyldimethylsilyl)-5-methyluridine (12)[S2]: 5-methyluridine **11** (3.25 gm, 12.59 mmol) was suspended in dry DMF (23 mL) and cooled in an ice bath. Di-*tert*-butylsilyl bis(trifluoromethanesulfonate) (4.5 mL, 13.84 mmol) was added dropwise and stirred for additional 1 h. To the above reaction imidazole (4.29 gm, 62.95 mmol) and TBS-Cl (2.28 gm, 15.10 mmol) were added and heated to 60°C for 2 h. Solvent was evaporated, resulting residue was dissolved in ethyl acetate (60 mL) and washed with water followed by brine (60 mL each). Organic phase was dried over anhydrous sodium sulphate, filtered, and evaporated. Crude product was purified by flash column chromatography (0−1 % methanol in dichloromethane) to afforded compound **12** as a white solid (5.87 gm, 91%).

5-Acetoxymethyl-3',5'-*O*-(di-*tert*-butylsilylene)-2'-*O*-(*tert*-butyldimethylsilyl)-uridine (13): To a solution of **12** (5.8 gm, 10.13 mmol) in dry benzene (60 mL) were added *N*-Bromosuccinimide (2.16 gm, 12.17 mmol) and AIBN (203 mg, 1.24 mmol) at 60 °C. The reaction mixture was refluxed for 40 min and cooled to room temperature, solvent was evaporated under reduced pressure. The crude residue was dissolved in dry DMF (30 mL) and then potassium acetate (2.00 gm, 20.30 mmol) was added. The resulting slurry was stirred at 50 °C for 30 min and diluted with ethyl acetate (100 mL). The organic solution was washed with water followed by brine (60 mL each), dried over anhydrous sodium sulfate, filtered and evaporated. Flash column chromatography (10−25 % ethyl acetate in hexane) afforded compound 13 (4.15 gm, 64 %) as a white solid. TLC (Hexane:EtOAc = 70:30); R_f = 0.50.**^{1}H-NMR (400 MHz, DMSO-d$_6$)**: δ (ppm) 11.59 (s, 1H), 7.68 (s, 1H), 5.68 (s, 1H), 4.76 (d, *J* = 9.2 Hz, 1H), 4.70 (d, *J* = 9.6 Hz, 1H), 4.42 (d, *J* = 4.0 Hz, 1H), 4.40 (d, *J* = 4.0 Hz, 1H), 4.09−4.05 (m, 1H), 3.97−3.92 (m, 2H), 1.98 (s, 3H), 1.03 (s, 9H), 0.99 (s, 9H), 0.89 (s, 9H), 0.13 (s, 3H), 0.09 (s, 3H).; **^{13}C-NMR (100 MHz, DMSO-d$_6$):** δ (ppm) 170.3, 162.6, 149.8, 141.1, 108.3, 92.8, 75.1, 74.3, 74.0, 66.8, 58.7, 27.3, 26.8, 25.7, 22.2, 20.7, 19.9, 18.0, -4.5, -5.1.; **HRMS (ESI+):** Calculated for $C_{26}H_{46}N_2O_8Si_2Na$ $[M+Na]^+$: 593.2690, found: 593.2693.

5-Hydroxymethyl-3',5'-*O*-(di-*tert*-butylsilylene)-2'-*O*-(*tert*-butyldimethylsilyl)-uridine (14): To a solution of **13** (4.0 gm, 7.00 mmol) in THF (20 mL) potassium carbonate (1.94 gm, 14.0 mmol) and water (8 mL) were added and stirred at room temperature for 4 h. Crude product was extracted in ethyl acetate and washed with water followed by brine (30 mL each). The organic phase was dried over anhydrous anhydrous sodium sulfate, filtered, and evaporated. Flash column chromatography (20−50 % ethyl acetate in hexane) afforded compound **14** (3.50 gm, 92%) as a white solid. TLC (Hexane:EtOAc = 60:40); R_f = 0.34.; **^{1}H-NMR (400 MHz, DMSO-d$_6$):** δ (ppm) 11.44 (s, 1H), 7.39 (s, 1H), 5.69 (s, 1H), 5.00 (t, *J* = 5.0 Hz, 1H), 4.41 (dd, *J* = 8.0 Hz, 4.5 Hz, 2H), 4.14 (d, *J* = 4.8 Hz, 2H), 4.05 (dd, *J* = 7.2 Hz, 4.0 Hz, 1H), 3.96−3.89 (m, 2H), 1.03 (s, 9H), 0.99 (s, 9H), 0.88 (s, 9H), 0.13 (s, 3H), 0.09 (s, 3H).; **^{13}C-NMR (100 MHz, DMSO-d$_6$):** δ (ppm) 162.6, 149.9, 136.6, 114.5, 92.9, 73.8, 66.9, 59.7, 55.7, 27.3, 26.8, 25.7, 22.2, 19.9, 18.0, -4.5, -5.2.; **HRMS (ESI+):** Calculated for $C_{24}H_{45}N_2O_7Si_2$ $[M+H]^+$: 529.2765, found: 529.2769

5-Formyl-3',5'-*O*-(di-*tert*-butylsilylene)-2'-*O*-(*tert*-butyldimethylsilyl)-uridine (15): To an ice cold solution of compound **14** (3.40 gm, 6.43 mmol) in dichloromethane (50 mL) Dess-Martin periodinane (3.27 gm, 7.72 mmol) was slowly added. Reaction was stirred at room temperature for 4 h and then quenched by adding saturated sodium carbonate and sodium thiosulfate solutions (15 mL each). Product was extracted in dichloromethane and washed

with water followed by brine (50 mL each). The organic phase was dried over anhydrous sodium sulfate, filtered and evaporated. Flash column chromatography (15–30 % ethyl acetate in hexane) afforded compound **15** (2.60 gm, 77%) as a white solid. TLC (Hexane:EtOAc = 70:30); R_f = 0.56.; **^{1}H-NMR (400 MHz, DMSO-d$_6$):** δ (ppm) 11.84 (s, 1H), 9.78 (s, 1H), 8.10 (s, 1H), 5.62 (s, 1H), 4.54 (d, *J* = 3.2 Hz, 1H), 4.50 (dd, *J* = 6.4 Hz, 3.2 Hz, 1H), 4.05–3.98 (m, 3H), 1.01 (s, 9H), 0.99 (s, 9H), 0.91 (s, 9H), 0.16 (s, 3H), 0.12 (s, 3H).; **^{13}C-NMR (100 MHz, DMSO-d$_6$):** δ (ppm) 186.4, 161.5, 149.2, 146.1, 110.7, 93.5, 74.7, 74.3, 67.0, 27.3, 26.7, 25.3, 22.2, 19.9, 17.9, -4.4, -5.0.; **HRMS (ESI+):** Calculated for $C_{24}H_{42}N_2O_7Si_2K$ [M+K]$^+$: 565.2168, found: 565.2178

5-(1,3-dioxan-2-yl)-3',5'-*O*-(di-*tert*-butylsilylene)-2'-*O*-(*tert*-butyldimethylsilyl)-uridine (16): To an ice-cold solution of Compound **15** (2.50 gm, 4.75 mmol) in dichloromethane (50 mL) 1,3-propanediol (1.36 mL, 18.98 mmol) and triethyl orthoformate (950 μL, 5.70 mmol) were added. Subsequently, 1M $TiCl_4$ solution (1.9 mL, 1.9 mmol) was slowly added to the reaction mixture and stirred for additional 1 h at 0 °C. Reaction was stopped by adding cold water (40 mL), separated organic layer was dried over anhydrous sodium sulfate, filtered and evaporated. Flash column chromatography (10-30% ethyl acetate in hexane) afforded compound **16** (2.45 gm, 88 %) as a white solid. TLC (Hexane:EtOAc = 60:40); R_f = 0.50.; **^{1}H-NMR (400 MHz, DMSO-d$_6$):** δ (ppm) 11.50 (s, 1H), 7.52 (s, 1H), 5.63 (s, 1H), 5.32 (s, 1H), 4.90 (d, *J* = 4.4 Hz, 2H), 4.07–4.00 (m, 4H), 3.92–3.98 (m, 3H), 1.95–1.85 (m, 1H), 1.39 (d, *J* = 12.4 Hz, 1H), 1.03 (s, 9H), 0.99 (s, 9H), 0.89 (s, 9H), 0.14 (s, 3H), 0.10 (s, 3H).; **^{13}C-NMR (100 MHz, DMSO-d$_6$):** δ (ppm) 161.4, 149.5, 138.3, 111.7, 94.7, 93.1, 74.8, 74.2, 74.0, 67.1, 66.8, 66.7, 27.3, 26.7, 25.7, 25.3, 22.4, 19.9, 18.0, -4.6, -5.1.; **HRMS (ESI$^+$):** Calculated for $C_{27}H_{49}N_2O_8Si_2$ [M+H]$^+$: 585.3027, found: 585.3035.

5-(1,3-dioxan-2-yl)-3',5'-*O*-(di-*tert*-butylsilylene)-2'-*O*-(*tert*-butyldimethylsilyl)-cytidine (17): Compound **16** (2.40 gm, 4.10 mmol) and DMAP (50 mg, 0.41 mmol) were dissolved in dry dichloromethane (24 mL) and triethylamine (5.7 mL). Trisyl chloride (1.87 gm, 3.21 mmol) was added in one portion and stirring was continued for 2 h at room temperature. Reaction mixture was diluted with dichloromethane (30 mL), washed with saturated sodium bicarbonate solution and brine (40 mL each), dried over sodium sulfate, filtered and evaporated. Next, crude product was dissolved in a mixture of THF (25 mL) and 32% aqueous ammonia (30 mL) and stirred for 1.5 h at room temperature. Final product was extracted in ethyl acetate (50 mL) and washed with saturated sodium bicarbonate and brine (50 mL each). Separated organic layer was dried over anhydrous sodium sulfate, filtered and evaporated. Flash column chromatography (2–4 % methanol in dichloromethane) afforded compound **17** as a white solid (1.60 gm, 67%). TLC (CH_2Cl_2:MeOH = 96:4); R_f = 0.4.; **^{1}H-NMR (400 MHz, DMSO-d$_6$):** δ (ppm) 7.62 (br, 1H), 7.55 (s, 1H), 6.70 (br, 1H), 5.66 (s, 1H) 5.37 (s, 1H), 4.43 (d, *J* = 4.0 Hz, 1H), 4.31 (d, *J* = 4.0 Hz, 1H), 4.09–4.01 (m, 3H), 3.97–3.85 (m, 4H), 2.02–1.97 (m, 1H), 1.40 (d, *J* = 10.4 Hz, 1H), 1.03 (s, 9H), 0.99 (s, 9H), 0.90 (s, 9H), 0.13 (s, 3H), 0.09 (s, 3H).; **^{13}C-NMR (100 MHz, DMSO-d$_6$):** δ (ppm) 162.7, 153.5, 140.4, 104.2, 97.1, 93.8, 75.1, 74.4, 73.7, 67.0, 66.6, 27.3, 26.8, 25.7, 25.1, 22.3, 19.9, 18.0, -4.6, -5.0.; **HRMS (ESI$^+$):** Calculated for $C_{27}H_{49}N_3O_7Si_2Na$ [M+Na]$^+$: 606.3007, found: 606.3012.

***N*^{4}-Acetyl-5-(1,3-dioxan-2-yl)-3',5'-*O*-(di-*tert*-butylsilylene)-2'-*O*-(*tert*-butyldimethyl-silyl)-cytidine (18):** Compound **17** (1.50 gm, 2.56 mmol) was dissolved in dry pyridine (23 mL) and cooled to 0 °C. Acetic anhydride (654 μL, 6.40 mmol) was slowly added to the

above solution, after addition cooling was removed and reaction mixture was stirred for 6 h at room temperature before quenched by methanol. Solvents were evaporated completely under reduced pressure and crude product was purified by flash column chromatography (0.5–3 % methanol in dichloromethane) to afforded compound **18** as a white solid (1.58 g, 95%). TLC (CH_2Cl_2:MeOH = 95:5); R_f = 0.8.; **^{1}H-NMR (400 MHz, DMSO-d$_6$):** δ (ppm) 9.27 (s, 1H), 7.88 (s, 1H), 5.65 (s, 1H) 5.62 (s, 1H), 4.47 (d, *J* = 3.6 Hz, 1H), 4.40 (d, *J* = 3.6 Hz, 1H), 4.16–4.03 (m, 4H), 4.00–3.89 (m, 3H), 2.40 (s, 3H) 1.99–1.90 (m, 1H), 1.50 (d, *J* = 11.2 Hz, 1H), 1.02 (s, 9H), 0.99 (s, 9H), 0.91 (s, 9H), 0.17 (s, 3H), 0.11 (s, 3H).; **^{13}C-NMR (100 MHz, DMSO-d$_6$):** δ (ppm) 170.7, 159.0, 152.9, 149.6, 144.8, 143.1, 123.9, 105.6, 97.4, 93.8, 74.8, 74.4, 74.2, 66.9, 66.8, 66.7, 27.3, 26.7, 25.7, 25.3, 22.2, 19.9, 17.9, -4.5, -5.0.; **HRMS (ESI+):** Calculated for $C_{29}H_{51}N_3O_8Si_2K$ $[M+K]^+$: 664.2852, found: 664.2859.

***N*^{4}-Acetyl-5-(1,3-dioxan-2-yl)-2'-*O*-(*tert*-butyldimethyl-silyl)-cytidine (19)** Compound **18** (1.55 gm, 2.48 mmol) was dissolved in anhydrous dichloromethane (15 mL) and cooled to 0 °C. Subsequently, a solution of 18 % HF•pyridine (1 mL, 9.60 mmol) was slowly added and reaction was stirred for additional 2 h. Solvents were evaporated and crude product was purified by flash column chromatography (2–5 % methanol in dichloromethane) to afforded compound **19** as a white solid (1.14 gm, 95%). TLC (CH_2Cl_2:MeOH= 95:5); R_f = 0.4.; ^{1}H-**NMR (400 MHz, DMSO-d$_6$):** δ (ppm) 9.16 (s, 1H), 8.58 (s, 1H), 5.67 (br, 1H) 5.43 (s, 1H), 5.23 (t, *J* = 4.0 Hz, 1H), 5.00 (d, *J* = 4.4 Hz, 1H), 4.16 (dd, *J* = 9.0 Hz, 2.0 Hz, 2H), 4.08 (t, *J* = 8.0 Hz, 1H), 3.93–3.87 (m, 5H), 3.62 (dd, *J* = 9.0 Hz, 3.4 Hz, 1H), 2.41 (s, 3H), 2.00–1.92 (m, 1H), 0.88 (s, 9H), 0.09 (s, 3H), 0.07 (s, 3H).; **^{13}C-NMR (100 MHz, DMSO-d$_6$):** δ (ppm) 170.1, 158.8, 153.3, 143.8, 136.1, 123.9, 105.1, 98.2, 90.5, 83.5, 76.4, 67.7, 66.9, 59.1, 25.7, 25.2, 18.0, -4.8, -4.9.; **HRMS (ESI+):** Calculated for $C_{21}H_{36}N_3O_8Si$ $[M+H]^+$: 486.2272, found: 486.2268.

***N*^{4}-Acetyl-5-(1,3-dioxan-2-yl)-5'-*O*-(4,4'-dimethoxytrityl)-2'-*O*-(*tert*-butyldimethylsilyl)-cytidine (19):** A solution of **18** (1.00 gm, 2.06 mmol), DMT-Cl (840 mg, 2.47 mmol) and DMAP (25 mg, 0.21 mmol) in anhydrous pyridine (7 mL) was stirred at room temperature under nitrogen atmosphere for overnight. Pyridine was evaporated under reduced pressure, resulting residue was diluted with dichloromethane (50 mL) and was washed with 5% sodium bicarbonate solution (30 mL) followed by brine (30 mL). The organic extract was dried over sodium sulphate, filtered and evaporated. Crude residue was purified by flash column chromatography (20–50 % ethyl acetate in hexane) to afford the product **19** as a white foam (1.03 g, 63%). (Hexane:EtOAc = 50:50); R_f = 0.50.; **^{1}H-NMR (400 MHz, DMSO-d$_6$):** δ (ppm) 9.28 (s, 1H), 8.16 (s, 1H), 7.42 (d, *J* = 6.0 Hz, 2H), 7.34–7.26 (m, 7H), 6.90 (dd, *J* = 7.2 Hz, 2.0 Hz, 4H), 5.81 (d, *J* = 3.5 Hz, 1H), 5.14 (d, *J* = 4.8 Hz, 1H), 4.89 (s, 1H), 4.21 (t, *J* = 3.0 Hz, 1H), 4.08–4.01 (m, 3H), 3.85 (dd, *J* = 9.2 Hz, 3.6 Hz, 1H), 3.79 (dd, *J* = 9.2 Hz, 3.6 Hz, 1H), 3.74 (s, 6H), 3.39–3.34 (m, 2H), 3.27 (dd, *J* = 8.8 Hz, 3.2 Hz, 1H), 2.40 (s, 3H), 1.75–1.66 (m, 1H), 1.23 (d, *J* = 10.0 Hz, 1H), 0.87 (s, 9H), 0.08 (s, 3H), 0.06 (s, 3H).; **^{13}C-NMR (100 MHz, DMSO-d$_6$):** δ (ppm) 170.6, 158.9, 158.2, 153.3, 144.6, 142.6, 135.4, 135.3, 129.7, 127.9, 127.7, 126.8, 113.2, 105.7, 97.6, 90.1, 85.9, 82.5, 76.4, 69.7, 66.5, 62.7, 59.7, 55.1, 25.7, 24.8, 20.8, 18.0, -4.8, -5.0.; **HRMS (ESI+):** Calculated for $C_{42}H_{54}N_3O_{10}Si$ $[M+H]^+$: 788.3578, found: 788.3577.

***N*^{4}-Acetyl-5-(1,3-dioxan-2-yl)-5'-*O*-(4,4'-dimethoxytrityl)-2'-*O*-(tert-butyldimethylsilyl)-cytidine-3'-*O*-(2-cyanoethyl-*N,N*-diisopro-pylphosphoramidite) (2):** To a solution of compound **19** (235 mg, 0.30 mmol) in anhydrous dichloromethane (2.5 mL) *N,N*-

diisopropylethylamine (260 μL, 1.49 mmol) was added and stirred for 5 min. To this solution 2-cyanoethyl *N,N*-diisopropylchlorophosphoramidite (106 μL, 0.45 mmol) was slowly added and the reaction mixture was stirred for 5 h. Solvent was evaporated to dryness, resulting residue was dissolved in ethyl acetate (20 mL), which was washed with 5% sodium bicarbonate solution followed by brine (15 mL each). Organic extract was dried over sodium sulphate, evaporated, filtered and crude residue was purified by flash column chromatography (25−55% ethyl acetate in hexane) to afford the product **2** as a white solid (230 mg, 76 %). TLC (EtOAc:Hexane = 25:75, few drops of triethylamine); R_f = 0.45.; **^{1}H-NMR (400 MHz, DMSO-d$_6$):** δ (ppm) 9.35 (s, 1H), 8.19 (s, 1H), 7.41 (d, *J* = 7.2 Hz, 2H), 7.42−7.26 (m, 7H), 6.90 (dd, *J* = 10.4 Hz, 4.8 Hz, 4H), 5.99 (d, *J* = 4.4 Hz, 1H), 4.99 (s, 1H), 4.43 (t, *J* = 4.0 Hz, 1H), 4.20 (br, 1H) 4.09 (dd, *J* = 9.8 Hz, 2.8 Hz, 1H), 3.82−3.76 (m, 3H), 3.74 (s, 6H), 3.57−3.38 (m, 6H), 3.20 (dd, *J* = 8.6 Hz, 2.8 Hz, 1H), 2.76 (dd, *J* = 7.4 Hz, 4.6 Hz, 1H), 2.38 (s, 3H), 1.72−1.60 (m, 1H), 1.23−1.17 (m, 2H), 1.08 (d, *J* = 5.6 Hz, 6H), 0.89 (d, *J* = 5.2 Hz, 3H), 0.84 (s, 9H), 0.04 (s, 6H).; **^{31}P-NMR (162 MHz, DMSO-d$_6$):** δ (ppm) 148.81, 148.54.; HRMS (ESI$^+$): Calculated for $C_{51}H_{71}N_5O_{11}PSi$ $[M+H]^+$: 988.4657, found: 988.4653.

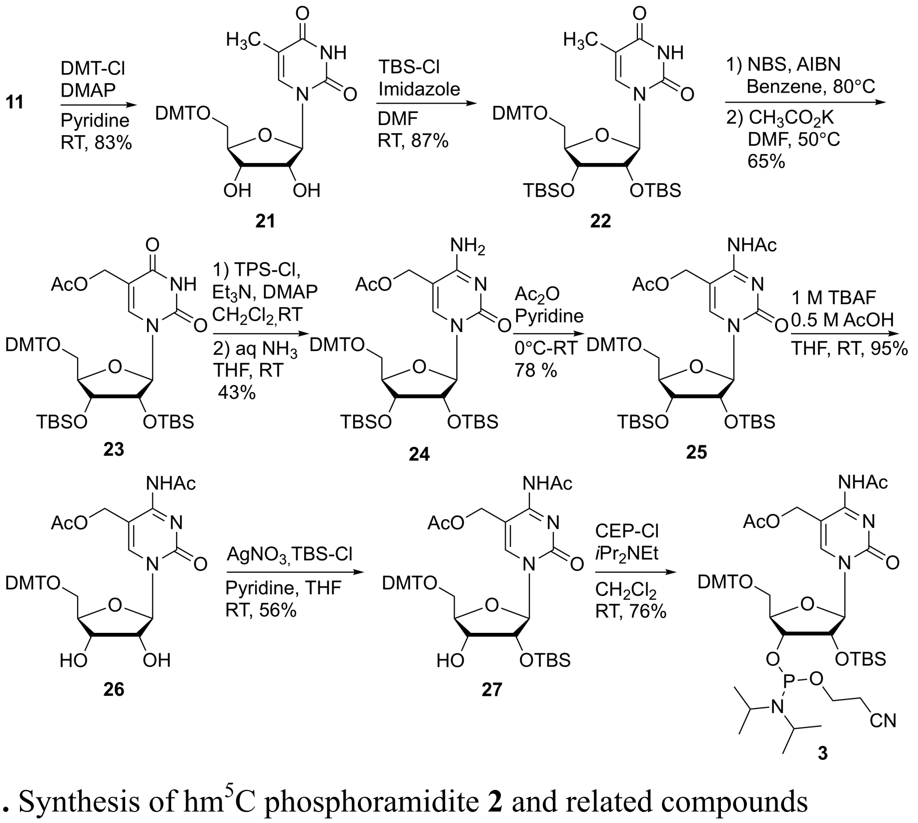

Scheme S3. Synthesis of hm^{5}C phosphoramidite **2** and related compounds

5'-*O*-(4,4'- dimethoxytrityl)-5-methyluridine (21)[S3]: A solution of 5-methyluridine **11** (2.00 gm, 7.75 mmol), DMT-Cl (3.28 gm, 9.68 mmol) and DMAP (10 mg, 0.80 mmol) in anhydrous pyridine (20 mL) was stirred at room temperature under nitrogen atmosphere for overnight. Pyridine was evaporated under reduced pressure. Crude mixture was then diluted with dichloromethane (50 mL) and washed with 5% sodium bicarbonate solution followed by brine (30 mL each). Organic extract was dried over sodium sulphate, evaporated and purified by flash column chromatography (0−5 % methanol in dichloromethane containing 0.5% triethylamine) to afford the product **21** as a white solid (3.62 gm, 83%).

5'-*O*-(4,4'-dimethoxytrityl)-2',3'-di-*O*-*tert*-butyldimethylsilyl-5-methyluridine (22): To a solution of compound **21** (3.6 gm, 6.42 mmol) in dry DMF (18 mL), Imidazole (2.19 gm, 32.1 mmol) and TBS-Cl (2.42 gm, 16.05 mmol) were added and the reaction was allowed to proceed for overnight at room temperature. All volatiles were evaporated under reduced pressure and residue was partitioned between ethyl acetate (100 mL) and water (70 mL). The organic layer was separated, washed with brine (60 mL), dried over sodium sulfate, filtered and evaporated. Crude product was purified by flash column chromatography (20–35% ethyl acetate in hexane) to yield the compound **22** as a white foam (4.42 gm, 87%). TLC (Hexane:EtOAc = 50:50); R_f = 0.5.; **^{1}H-NMR (400 MHz, DMSO-d$_6$):** δ (ppm) 11.43 (s, 1H), 7.60 (s, 1H), 7.40 (d, *J* = 7.6 Hz 2H), 7.34–7.24 (m, 7H), 6.90 (dd, *J* = 8.8 Hz, 2.0 Hz, 4H), 5.80 (d, *J* = 5.6 Hz, 1H), 4.36 (t, *J* = 5.2 Hz, 1H), 4.06 (t, *J* = 4.8 Hz, 1H), 4.00–3.98 (m, 1H), 3.74 (s, 6H), 3.37 (dd, *J* = 11.2 Hz, 2.8 Hz, 1H), 3.19 (dd, *J* = 11.2 Hz, 2.8 Hz, 1H), 1.46 (s, 3H), 0.83 (s, 9H), 0.78 (s, 9H), 0.02 (s, 3H), 0.01 (s, 3H), 0.00 (s, 3H), -0.06 (s, 3H).; **^{13}C-NMR (100 MHz, DMSO-d$_6$):** δ (ppm) 163.6, 158.4, 150.7, 144.6, 135.4, 135.0, 129.8, 128.0, 127.6, 127.0, 113.4, 109.9, 87.4, 86.5, 83.8, 74.6, 72.0, 63.0, 59.8, 55.2, 25.7, 25.6 17.7, 11.9, -4.5, -4.8, -4.9, -5.0.; **HRMS (ESI+):** Calculated for $C_{43}H_{60}N_2O_8Si$ $[M+H]^+$: 788.3888, found: 788.3889.

5'-*O*-(4,4'-dimethoxytrityl)-2',3'-di-*O*-*tert*-butyldimethylsilyl-5-acetyloxy-methyluridine (23)[S4]: To a solution of **22** (4.4 gm, 5.57 mmol) in dry benzene (66 mL), N-bromo-succinimide (1.19 gm, 6.69 mmol) and AIBN (110 mg, 0.67 mmol) were added at 60 °C. Reaction mixture was refluxed for 40 min before cooled to room temperature. Solvent was evaporated under reduced pressure. Crude bromo-derivative was dissolved in dry DMF (20 mL) and then potassium acetate (1.1 gm, 11.14 mmol) was added, resulting slurry was stirred at 50 °C for 30 min. Reaction mixture was diluted with ethyl acetate (50 mL) and washed with water followed by brine (40 mL each), dried over anhydrous sodium sulfate, filtered and evaporated. Flash column chromatography purification (10–20% ethyl acetate in hexane) afforded compound **23** (3.07 gm, 65%) as a white solid. For NMR see S4.

5-Acetyloxymethyl-5'-*O*-(4,4'-dimethoxytrityl)-2'-3'-di-*O*-*tert*-butyldimethylsilylcytidine (24) [S4]: Compound **23** (3.00 gm, 3.54 mmol) and DMAP (32 mg, 0.27 mmol) were dissolved in dry dichloromethane (20 mL) and triethylamine (5.0 mL, 35.4 mmol). Trisyl chloride (1.6 gm, 5.31 mmol) was added in one portion and stirring was continued for 2 h at room temperature. Reaction mixture was diluted with dichloromethane (30 mL), washed with saturated sodium bicarbonate solution and brine (50 mL each), dried over anhydrous sodium sulfate, filtered and evaporated. Next, the crude product was dissolved in a mixture of THF (50 mL) and 32% aqueous ammonia (70 mL) and stirred for additional 2 h at room temperature. Final product was extracted in ethyl acetate (60 mL) and washed with saturated sodium bicarbonate and brine (70 mL each), dried over anhydrous sodium sulfate, filtered and evaporated. Flash column chromatography (1–5 % methanol in dichloromethane) provided compound **24** as a white solid (1.29 gm, 43%).

***N^4*-Acetyl-5-acetyloxymethyl-5′-*O*-(4,4′-dimethoxytrityl)-2′,3′-di-*O*-*tert*-butyldimethyl-silylcytidine (25) [S4]**: Compound **24** (1.2 gm, 1.42 mmol) was dissolved in dry pyridine (25 mL) and cooled to 0 °C. Acetic anhydride (365 μL, 3.55 mmol) was slowly added to the above solution, after addition cooling was removed and reaction was stirred at room temperature for additional 4 h before quenched by methanol. Solvents were evaporated completely under reduced pressure and crude product was purified by flash column

chromatography (0−3 % methanol in dichloromethane) to afforded compound **25** as a white solid (983 mg, 78%).

***N⁴*-Acetyl-5-acetyloxymethyl-5'-*O*-(4,4'-dimethoxytrityl) cytidine (26)** [S4]**:** Compound **25** (970 mg, 1.09 mmol) was dissolved in a solution of 1 M TBAF (6 mL) containing 0.5 M AcOH in THF and stirred at 35 °C for 2 h. Solvents were evaporated and the residue was dissolved in dichloromethane (30 mL), washed with water followed by brine (20 mL each). The organic layer was dried over anhydrous sodium sulfate, filtered and evaporated. Crude product was purified by flash column chromatography (1−5 % methanol in dichloromethane) to afforded compound **26** as a white solid (684 mg, 95%).

***N⁴*-Acetyl-5-acetyloxymethyl-5′-*O*-(4,4′-dimethoxytrityl)-2′-*O*-tert-butyldimethylsilyl-cytidine (27)** [S4]: Compound **26** (670 mg, 1.02 mmol) and silver nitrate (381 mg, 2.24 mmol) were dissolved in anhydrous pyridine (2 ml). To the above solution was added anhydrous THF (9 ml). After stirring for 10 min *tert*-butyldimethylsilyl chloride (338 mg, 2.24 mmol) was added. Reaction mixture was stirred for additional 40 min and filtered through celite pad. Celite pad was washed with ethyl acetate (15 ml, twice) and the combined organic solution was then washed with 5 % sodium bicarbonate followed by brine (25 ml each). The organic extract was dried over sodium sulphate, filtered and evaporated. The resulting crude residue was purified by flash column chromatography to afford the product **27** as a white solid (440 mg, 56%).

***N⁴*-Acetyl-5-acetyloxymethyl-5′-*O*-(4,4′-dimethoxytrityl)-2′-*O*-*tert*-butyldimethylsilyl-cytidine-3′-*O*-(2-cyanoethyl-N,N-diisopropylphosphoramidite) (3)**[S4]: To a solution of compound **27** (300 mg, 0.39 mmol) in anhydrous dichloromethane (3 mL) was added *N,N*-diisopropylethylamine (0.340 µL, 1.94 mmol) and stirred for 5 min. To this solution 2-cyanoethyl *N,N*-diisopropylchlorophosphoramidite (140 µL, 0.59 mmol) was slowly added and reaction mixture was stirred for 5 h. Solvent was evaporated to dryness and residue was dissolved in ethyl acetate (20 mL), which was washed with 5% sodium bicarbonate followed by brine (15 mL each). Organic extract was dried over anhydrous sodium sulphate, filtered and evaporated. Crude residue was purified by flash column chromatography (25−55% ethyl acetate in hexane containing 1% triethylamine) to afford the product **3** as a white solid (287 mg, 76 %).

3. Solid-Phase RNA ON Synthesis.

Cytosine-modified RNA **1−4** were synthesised on a 1µmol scale CPG solid support (1000Å) by following a standard solid-phase RNA ON synthesis protocol.[S5] Incorporation of regular 2′-O-TBS-protected monomers was performed with a coupling time of 6 min; incorporation of cytosine-modified monomers **1−3** was performed with a coupling time of 10 min. The trityl protecting group was deprotected on the synthesiser. The solid support was treated with a solution of 20 % Ethanol in NH_4OH (1 mL) for 6 h at RT. The mixture was centrifuged, and supernatant was evaporated to dryness in a SpeedVac. The residue was then dissolved in 1:1 mixture of anhydrous DMSO and TEA·3HF (200 µL). The sample was heated at 65 ºC for 2.5h and was brought to RT. TBS-deprotected ONs where precipitated by adding 3M NH_3OAc (20 µL), and n-butanol (3 mL) and further isolation was carried out by storing samples at –80 °C for 30 mins follwed by centrifugation at 4 °C for 10 mins. RNA ONs **1** and **3** were purified by HPLC at this stage. For acetal deprotection RNA ONs **2** and **4** were dissolved in 20 % acetic acid (100 µL) and incubated at RT for 6 h. Samples then passed through gel filtration column prior to HPLC purifications.

4. LC-MS analysis of RNA ONs

LC-MS analysis of RNA ONs was performed on a Bruker amazon system, using an XTerra MS C18 column (2.5 µM, 2.1 x 50 mm), using solvents A (100 mM 1,1,1,3,3,3-Hexafluoro-2-propanol, 10 mM NEt_3) and B (MeOH). Flow-rate was kept at 0.2 mL/min with a gradient of 5–30% B in 25 min. Integrity of all RNA ONs was confirmed by ESI-MS signal at their respective retention time. See figure S1 for chromatogram mass data.

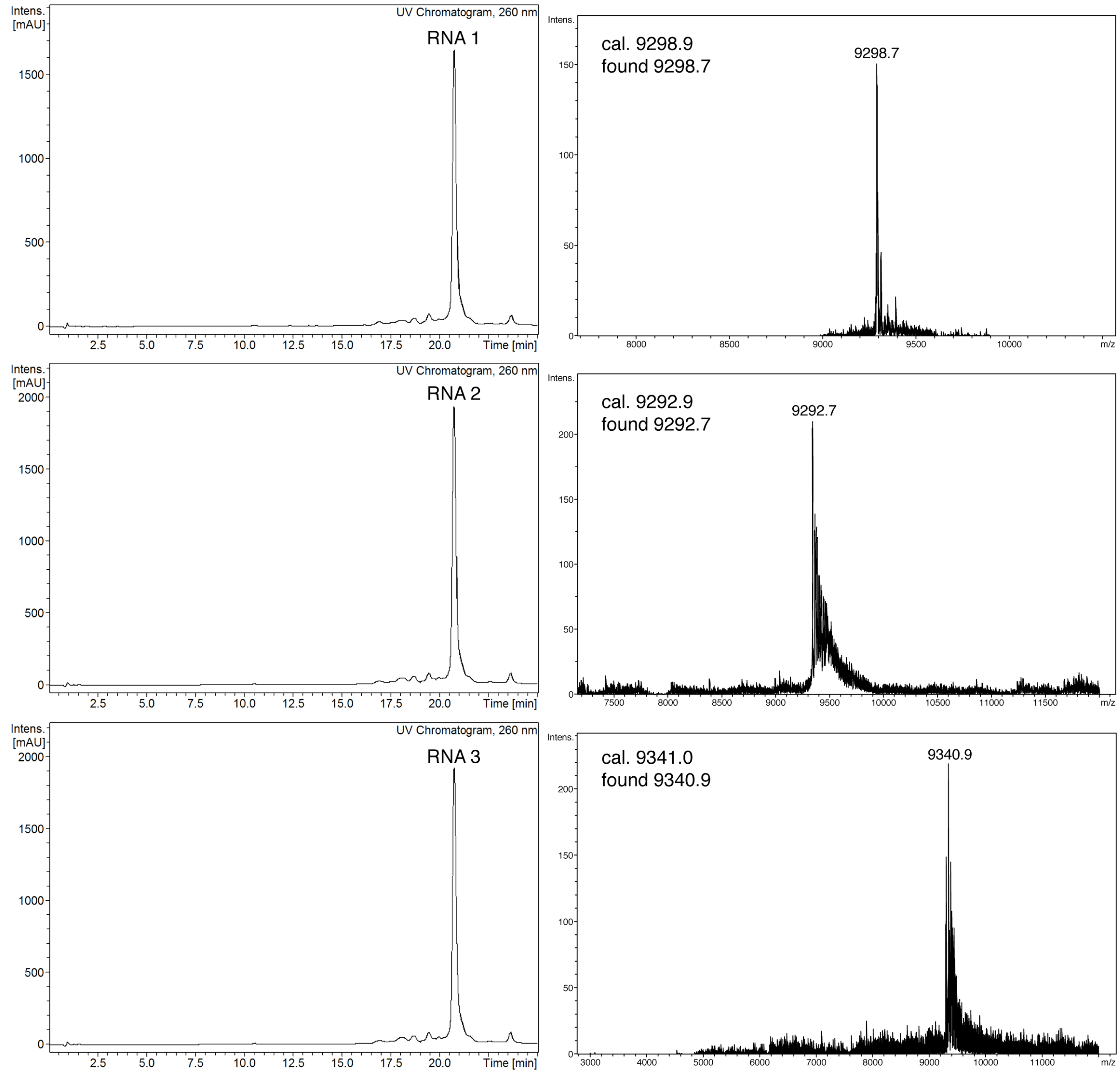

Figure S1. LC trace of RNA ONs **1–3** after HPLC-purification at 260 nm (Left). Corresponding ESI-MS spectrum (right)

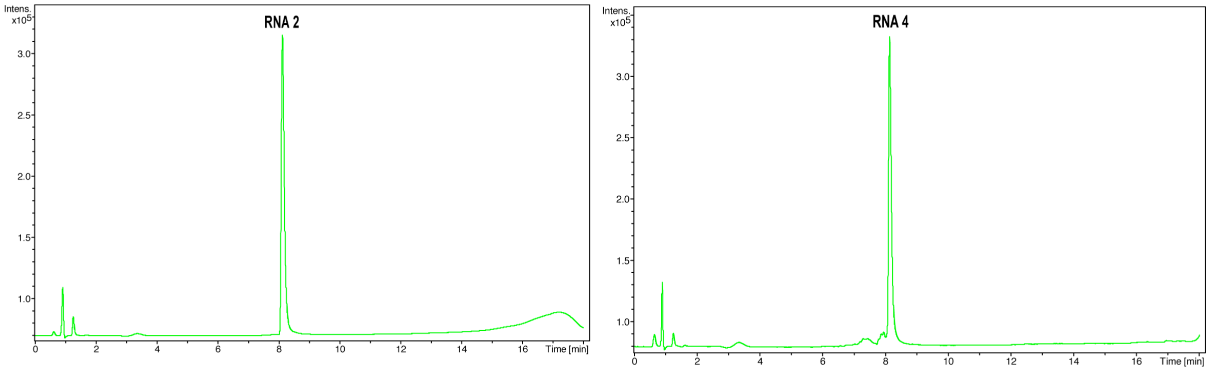

Figure S2. HPLC chromatograms of RNA ONs **2** and **4** after acetal deprotection.

5. Postsynthetic chemical functionalisation of RNA ONs

RNA ONs **1/2/3** (5 µl, 0.1 mM) were mixced in ammonium acetate buffer pH 5.0 (5 µl, 1 M), water (30 µl) and to these solutions p-anisidine (5 µl, 1 M in methanol) and ethoxy amine hydrochloride (5 µl, 100 mM) were added. The individual reaction (total volume 50 µl containing 10 % methanol) were incubated at 37 °C for overnight.[S6] All the samples then passed through gel filtration column prior to LC-MS analysis. Only RNA ON **2**, which contains reactive formyl group undergoes condensation reaction and forms a desired product, while RNA ONs **1** and **3** remain intact during this reaction.

6. References

S1. A. F. Lewis, G. R. Revankar, M. E. Hogan, *J. Heterocycl. Chem.* **1993**, *30*, 1309–1315.

S2. A. L. Allan, P. L. Gladstone, M. L. P. Price, S. A. Hopkins, J. C. Juarez, F. Doñate, R. J. Ternansky, D. E. Shaw, B. Ganem, Y. Li, W. Wang, S. Ealick, *J. Med. Chem.* **2006**, *49*, 7807–7815.

S3. A-F. Maggio, V. Boyer, A-M. Aubertin, G. Obert, A. Kirn, J-L. Imbach, *Nucleosides & Nucleotides* **1991**, *10*, 1431–1449.

S4. C. Riml, R. Micura, *Synthesis* **2016,** *48*, A–I.

S5. B. S. Sproat, *Methods in Molecular Biology*, *Oligonucleotide Synthesis* **2005**, *288*, 17–32.

S6. E.-A. Raiber, D. Beraldi, G. Ficz, H. E. Burgess, M. R. Branco, P. Murat, D. Oxley, M. J. Booth, W. Reik, S. Balasubramanian, *Genome Biol.* 2012, *13*, R69.

7. NMR spectra

^{1}H NMR of compound **6** in DMSO-d$_6$

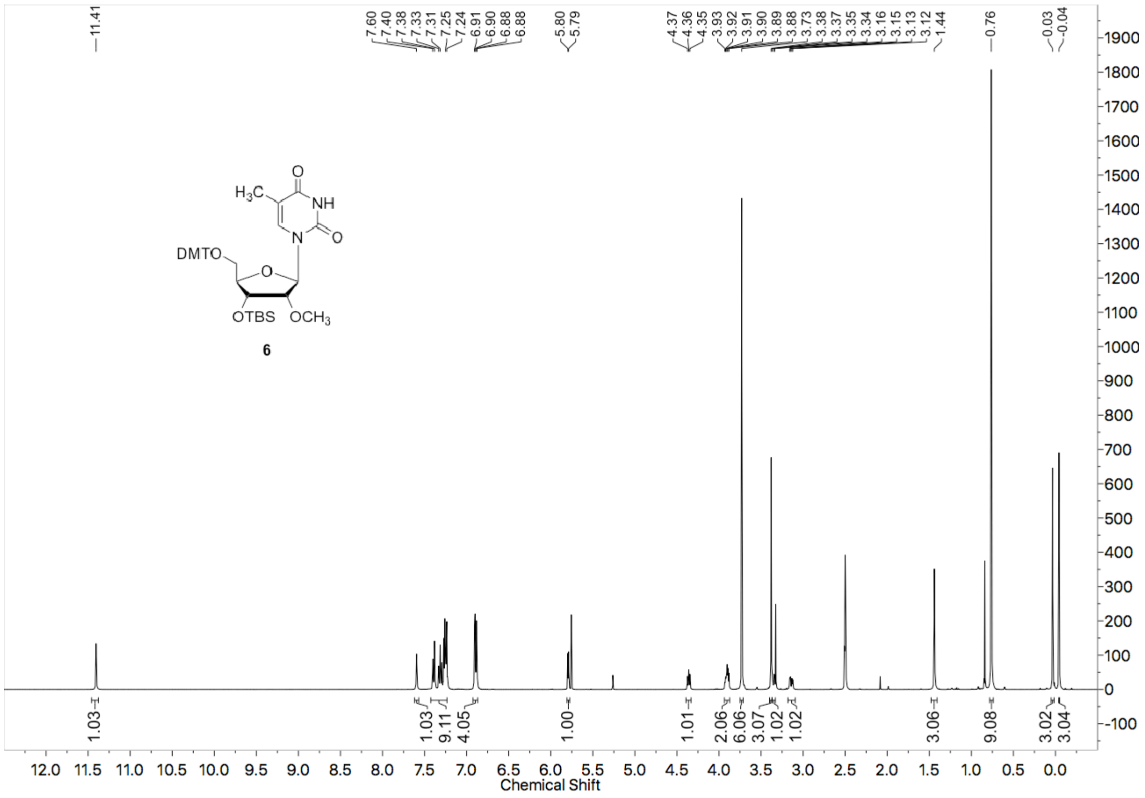

^{13}C NMR of compound **6** in DMSO-d$_6$

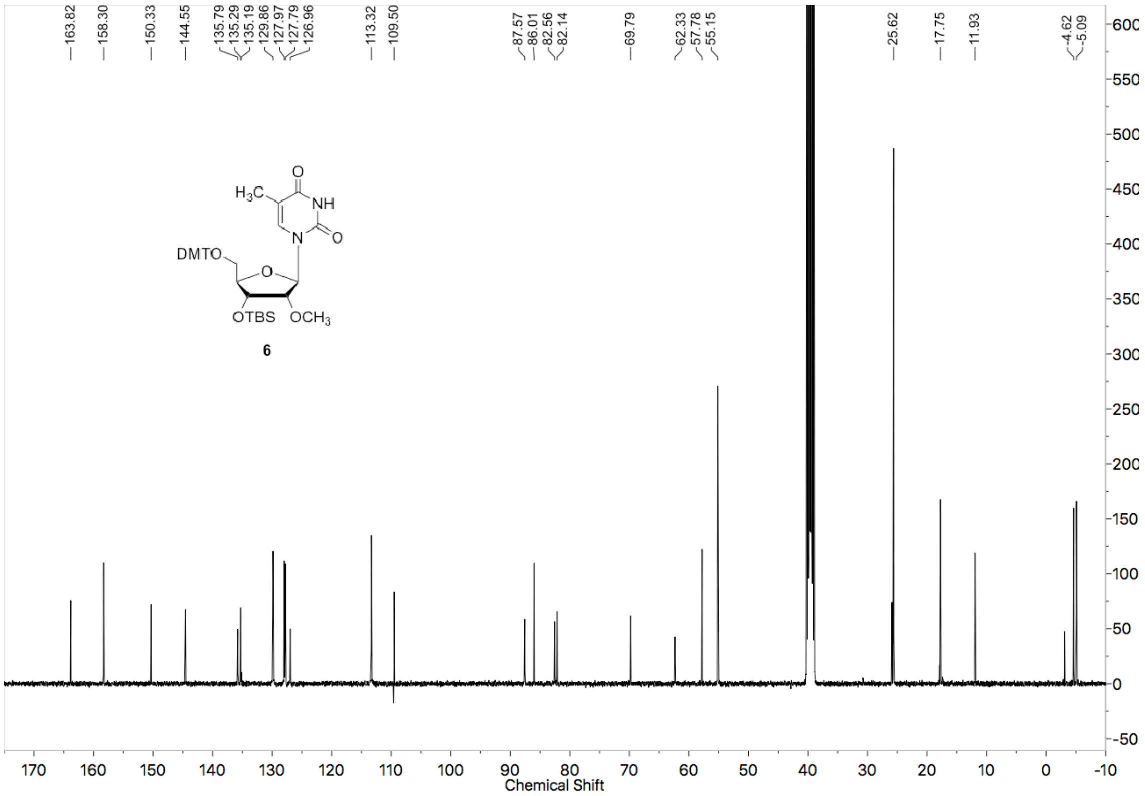

^{1}H NMR of compound **7** in DMSO-d$_6$

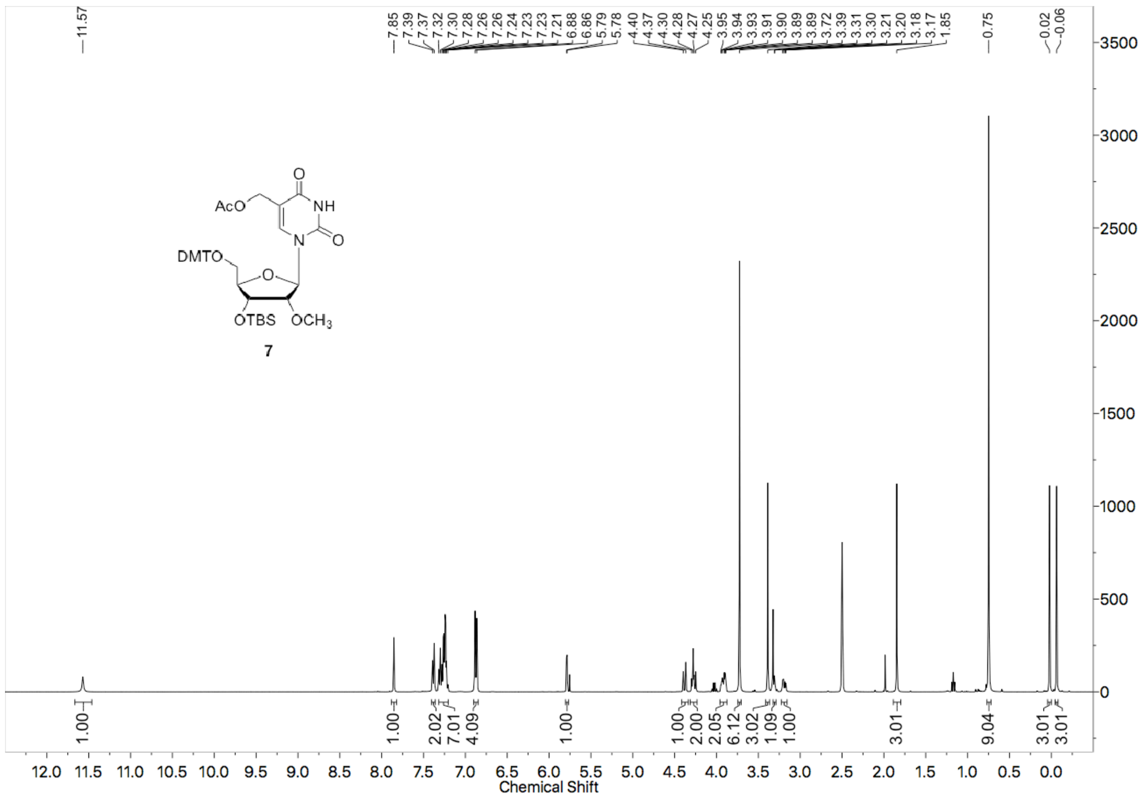

^{13}C NMR of compound **7** in DMSO-d$_6$

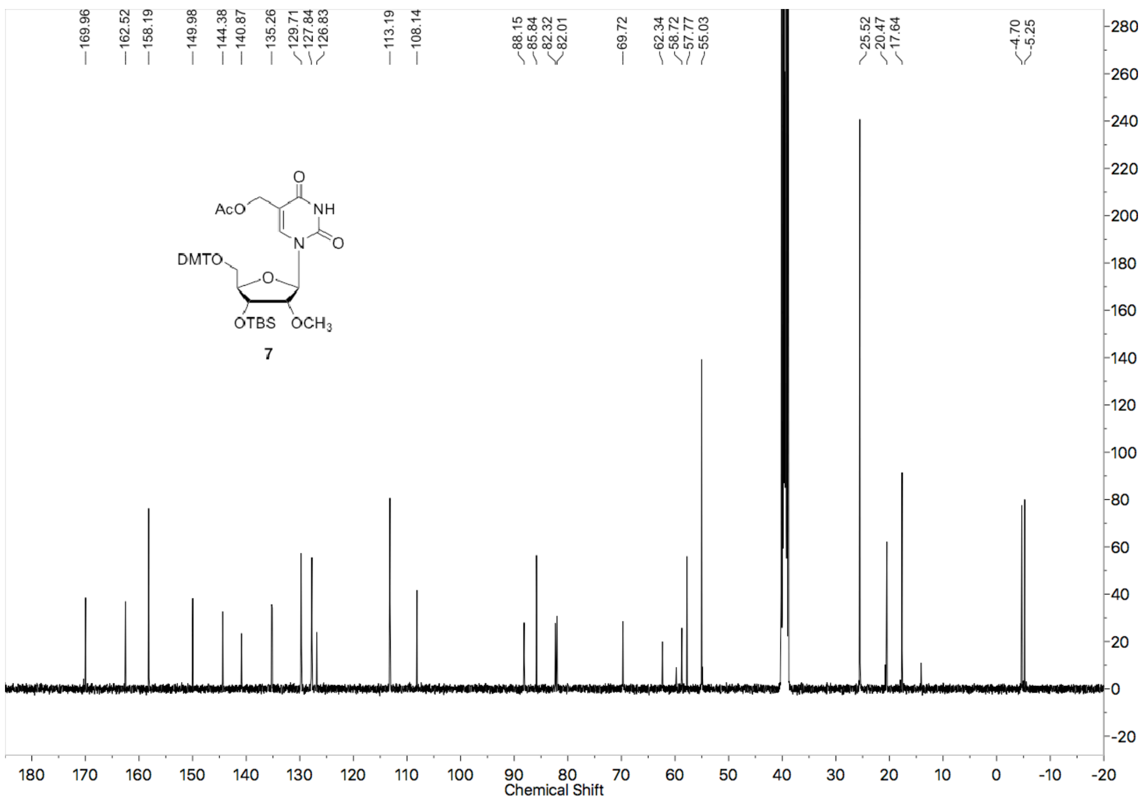

^{1}H NMR of compound **8** in DMSO-d_6

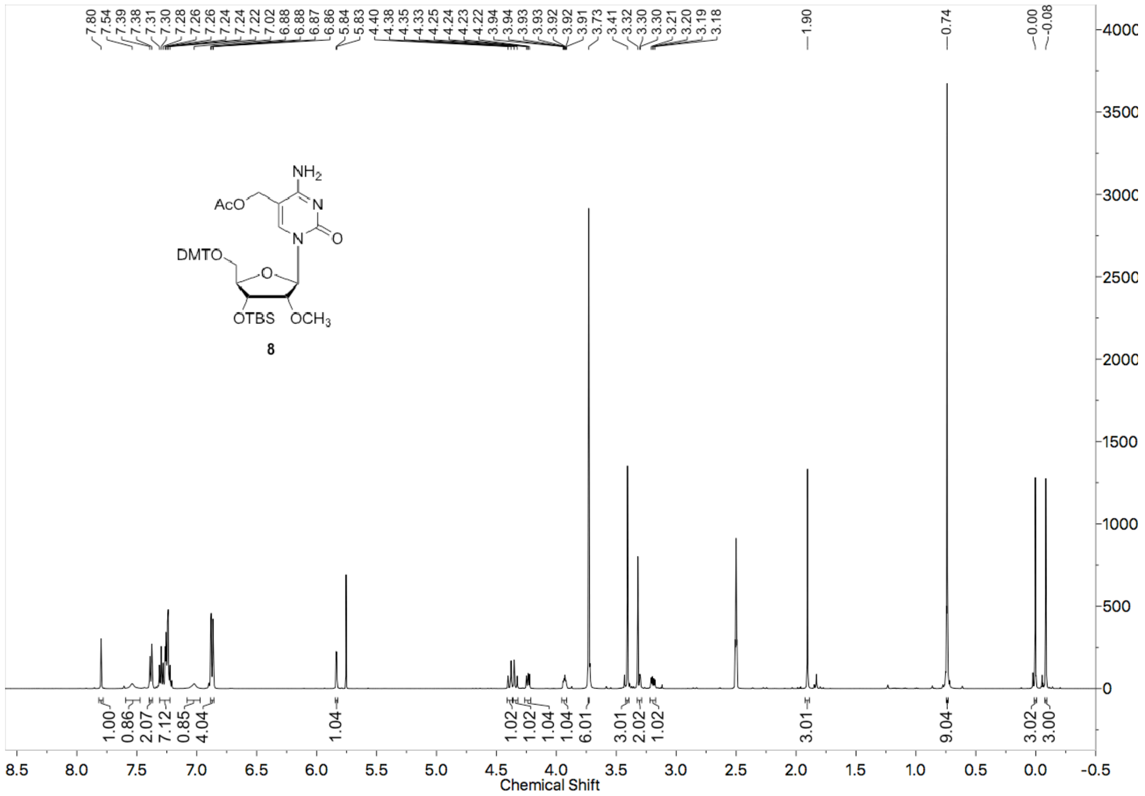

^{13}C NMR of compound **8** in DMSO-d_6

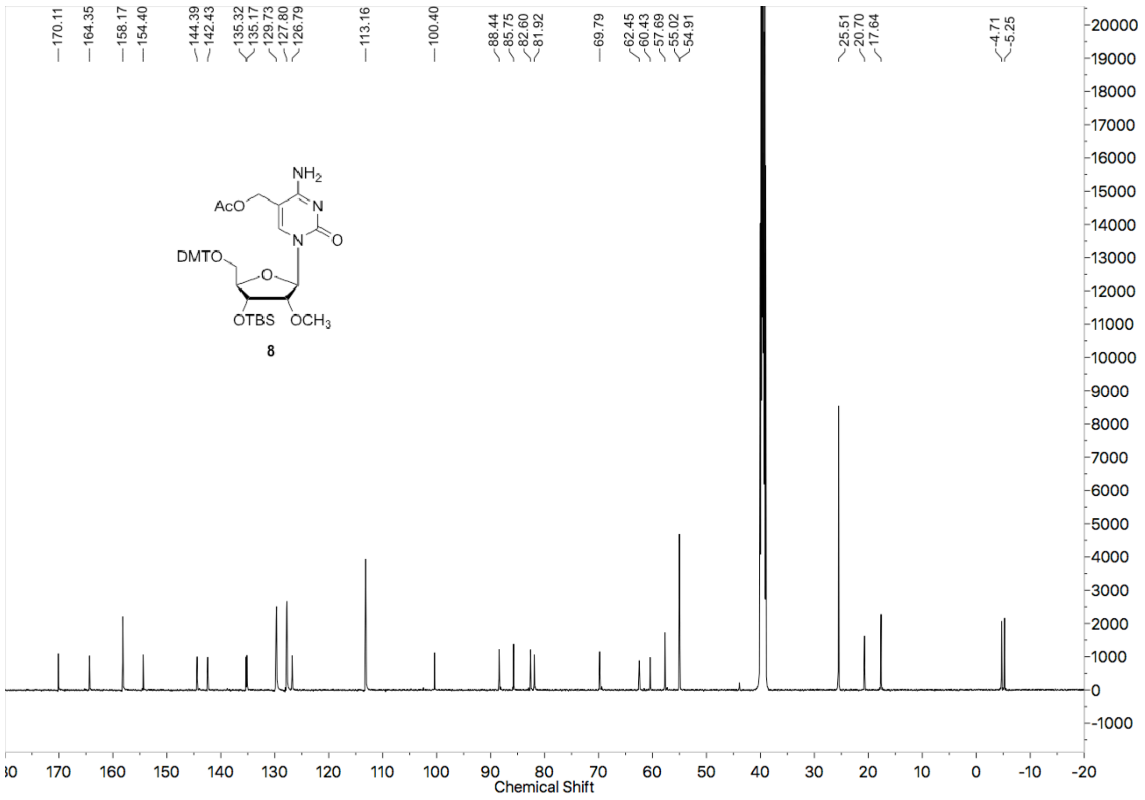

^{1}H NMR of compound **9** in DMSO-d$_6$

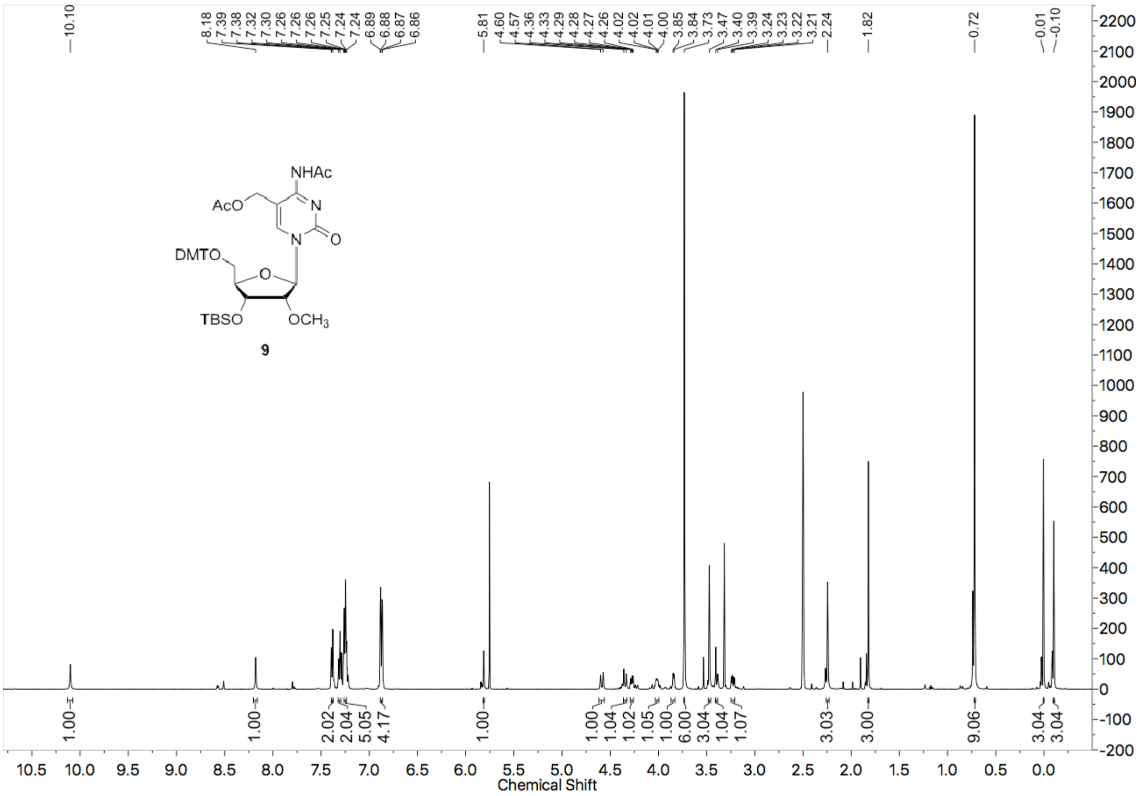

^{13}C NMR of compound **9** in DMSO-d$_6$

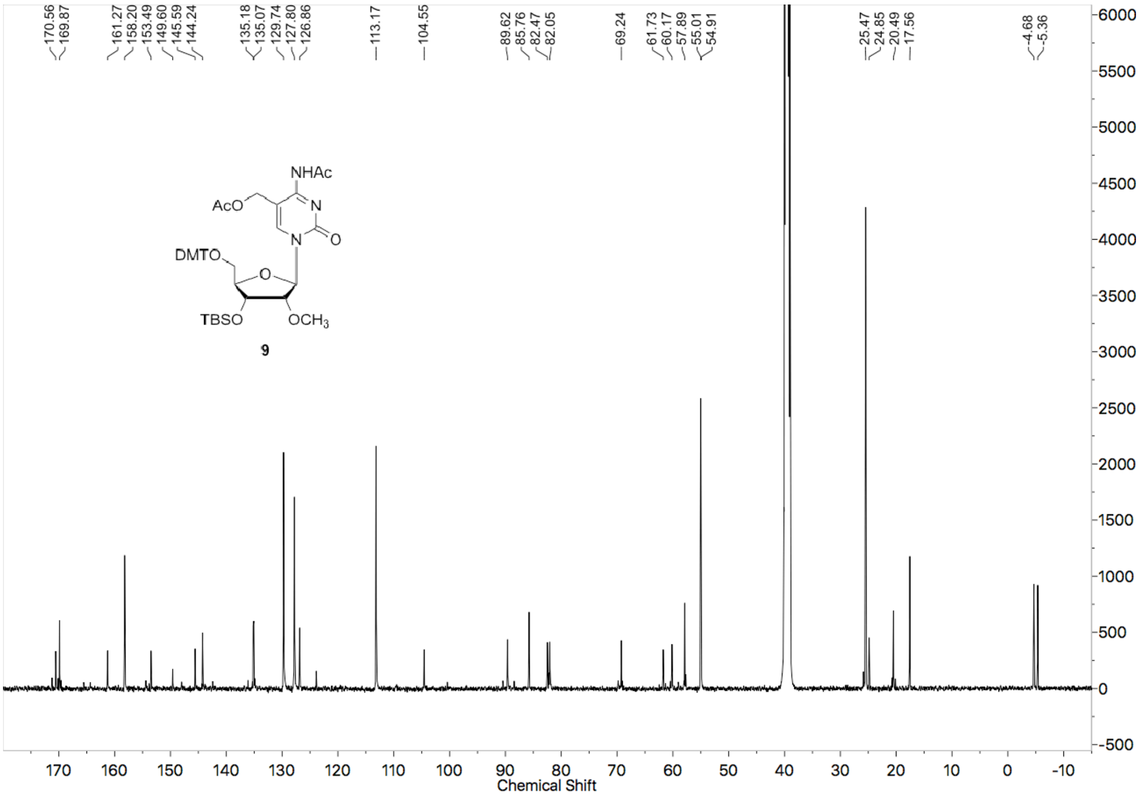

^{1}H NMR of compound **10** in DMSO-d_6

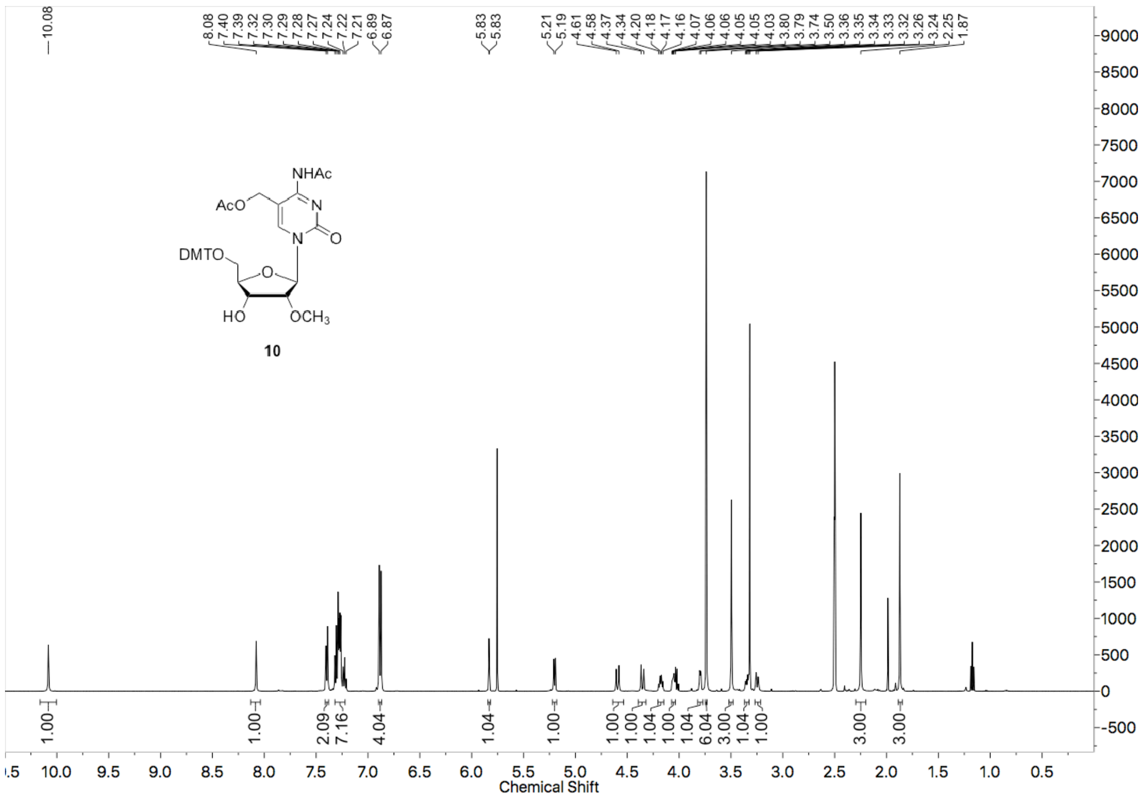

^{13}C NMR of compound **10** in DMSO-d_6

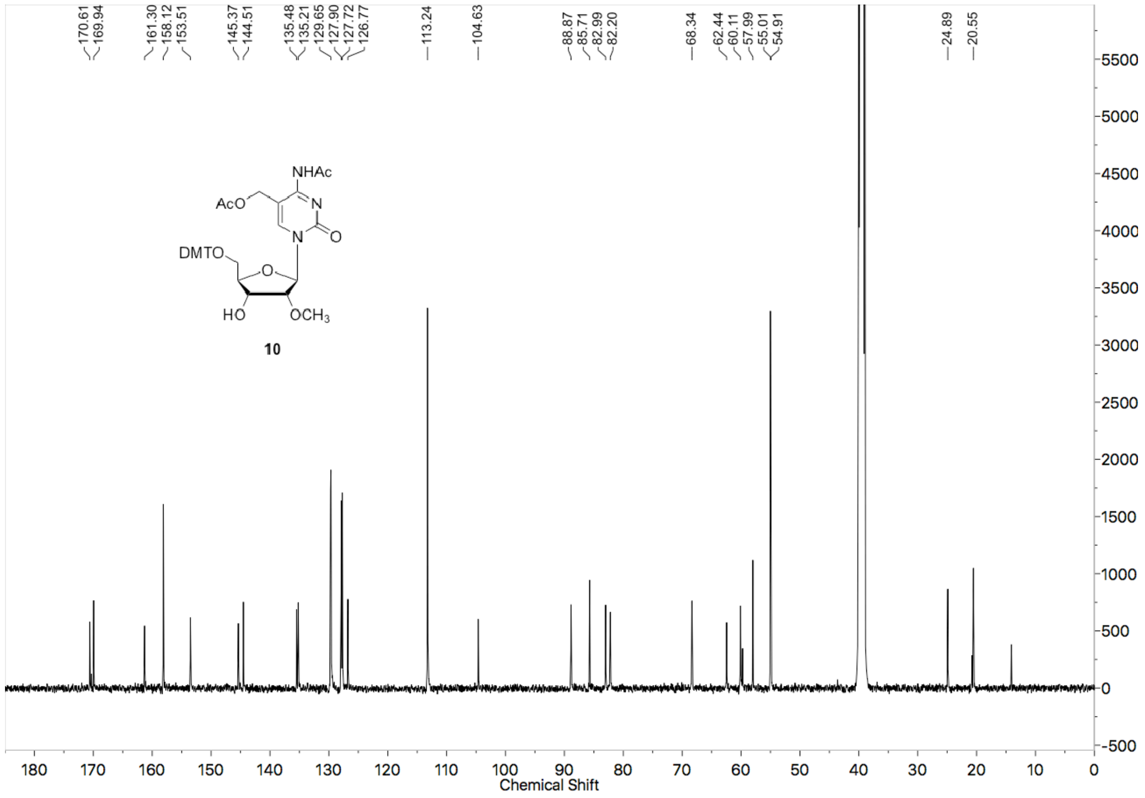

^{1}H NMR of phosphoramidite monomer **1** in DMSO-d_6

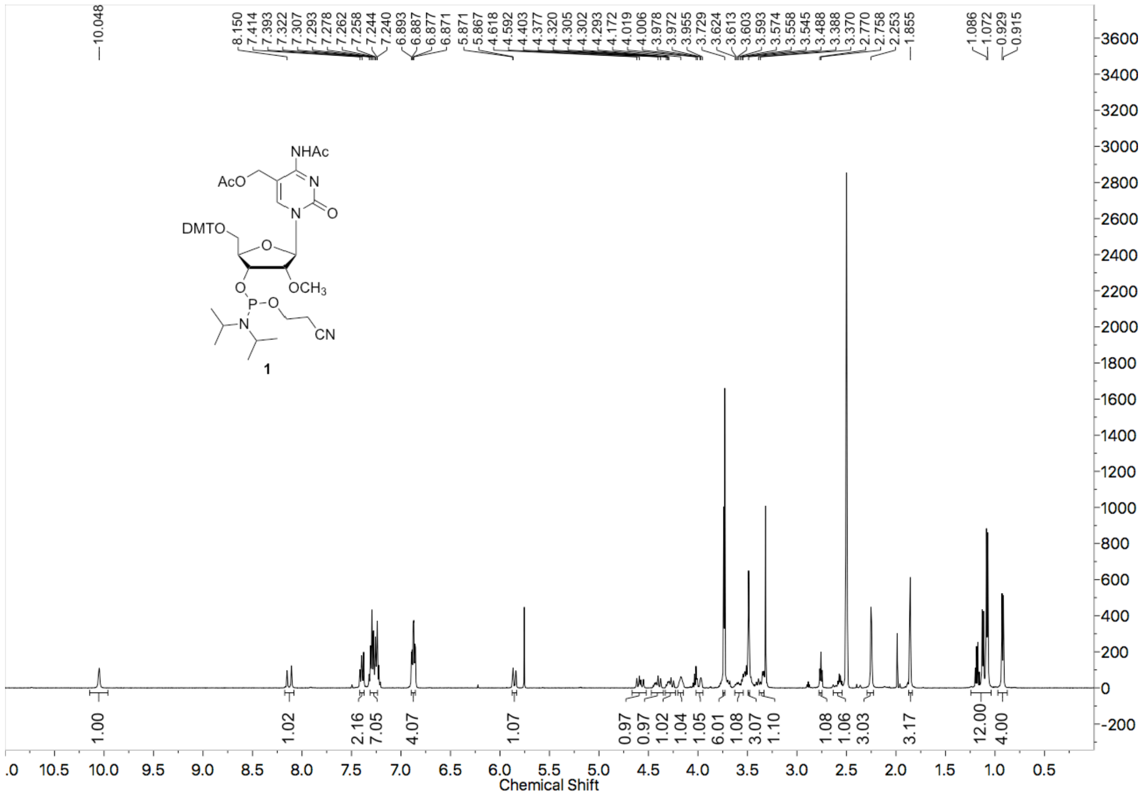

^{31}P NMR of phosphoramidite monomer **1** in DMSO-d_6

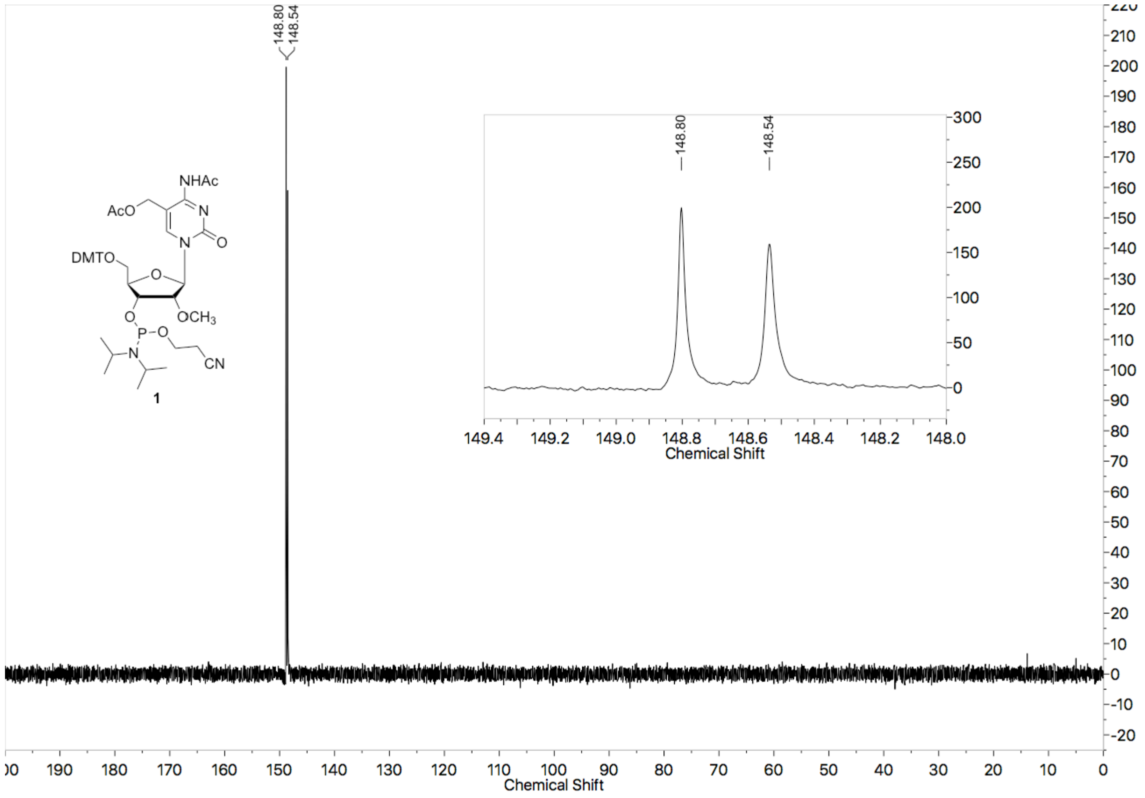

^{1}H NMR of compound **13** in DMSO-d_6

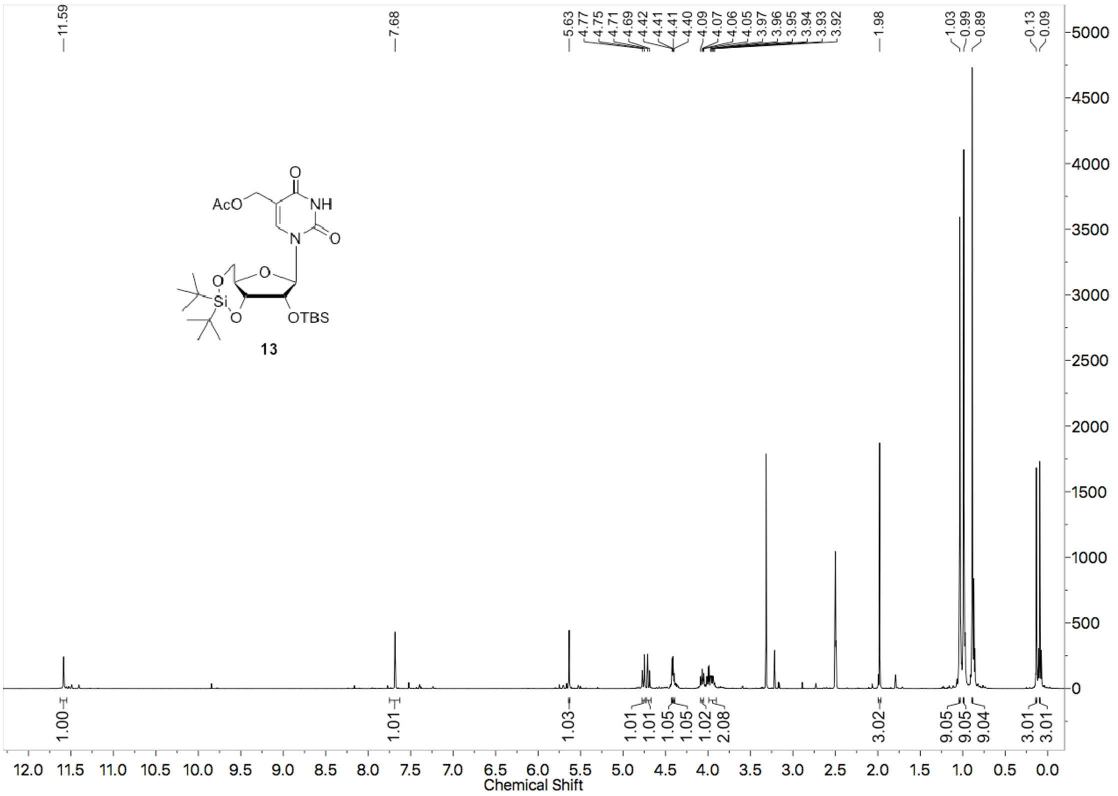

^{13}C NMR of compound **13** in DMSO-d_6

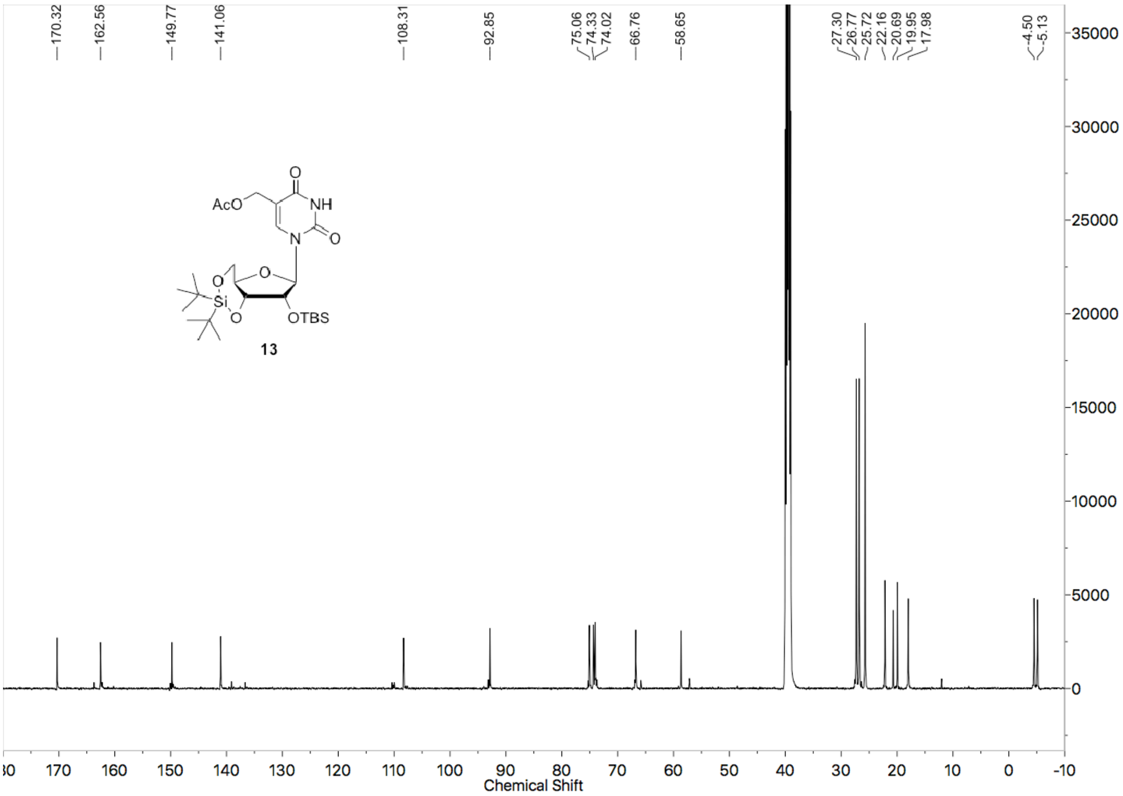

^{1}H NMR of compound **14** in DMSO-d_6

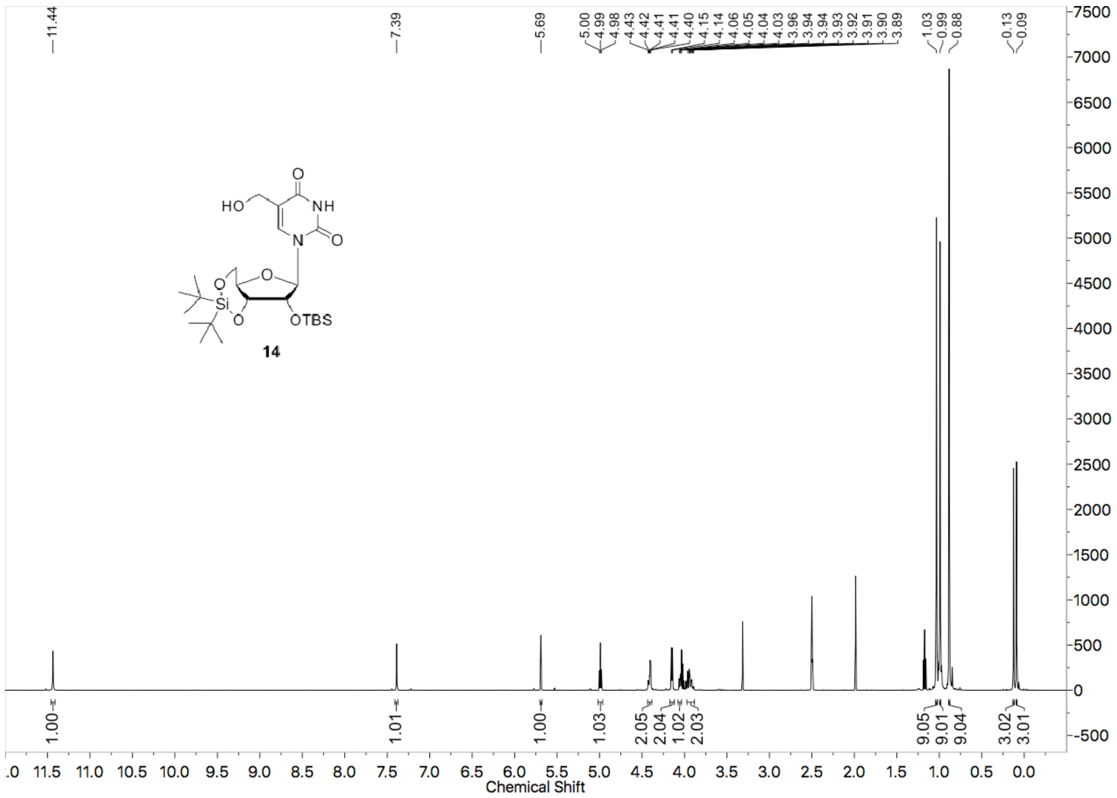

^{13}C NMR of compound **14** in DMSO-d_6

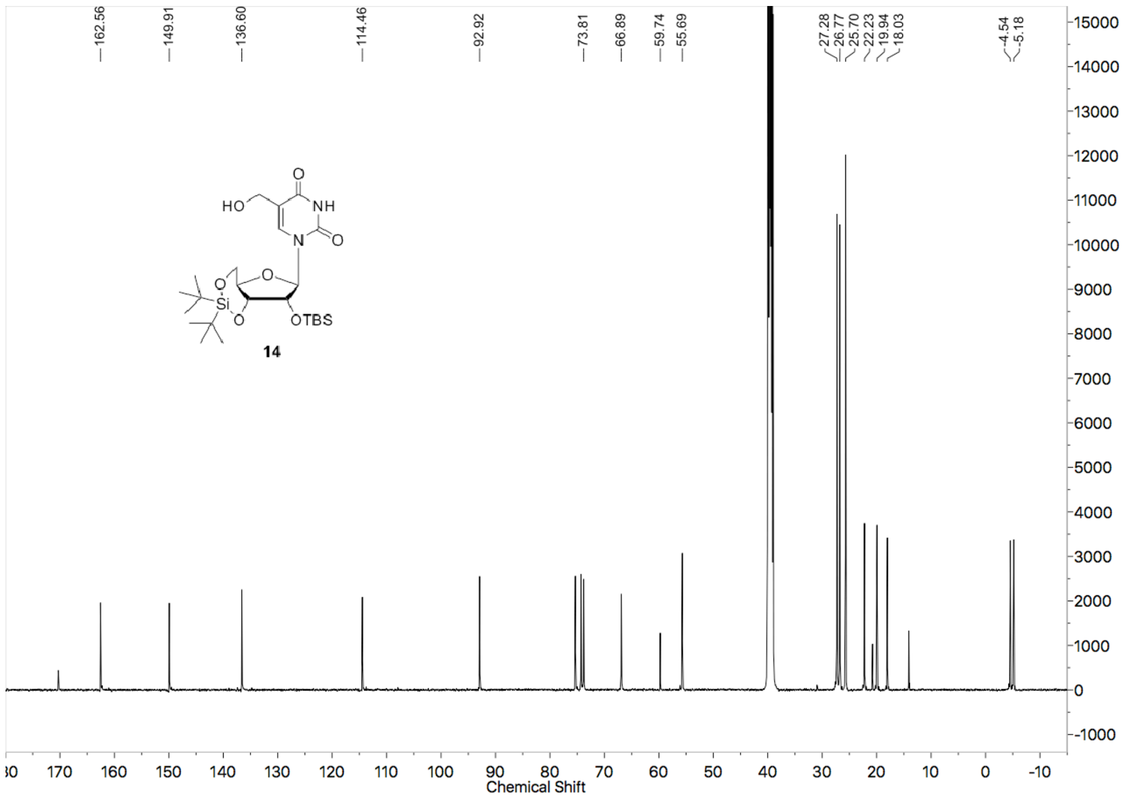

^{1}H NMR of compound **15** in DMSO-d$_6$

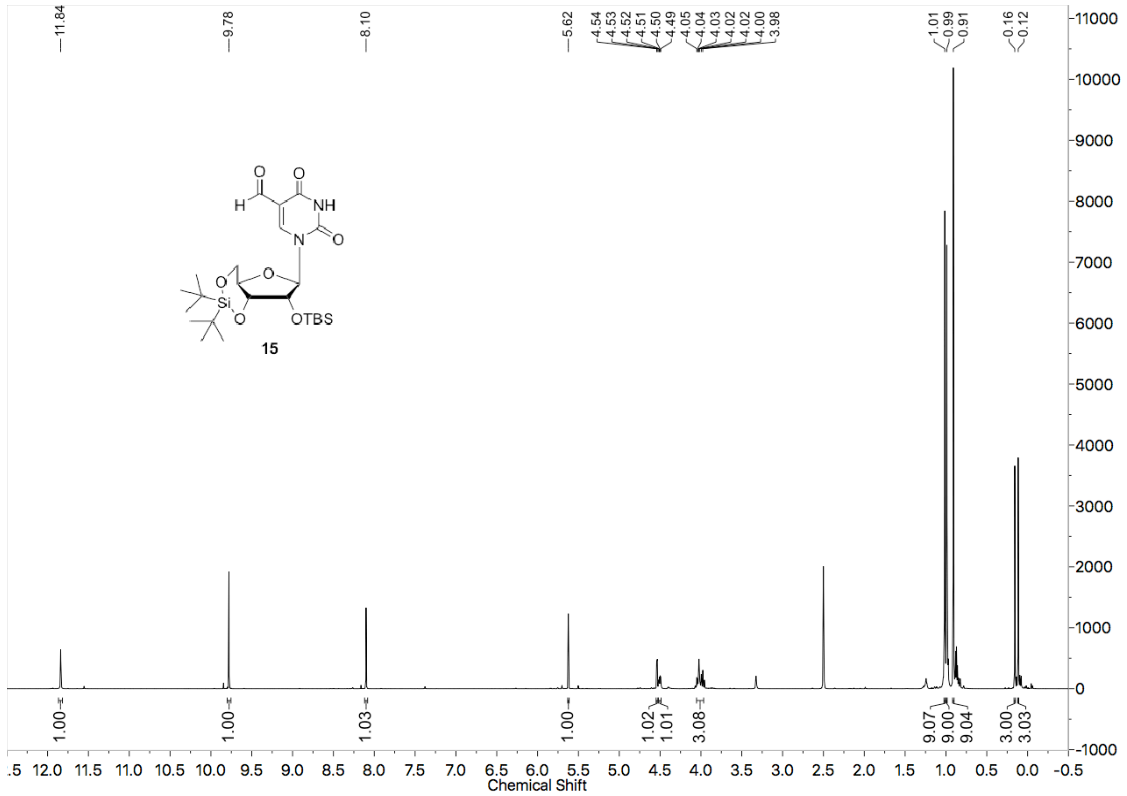

^{13}C NMR of compound **15** in DMSO-d$_6$

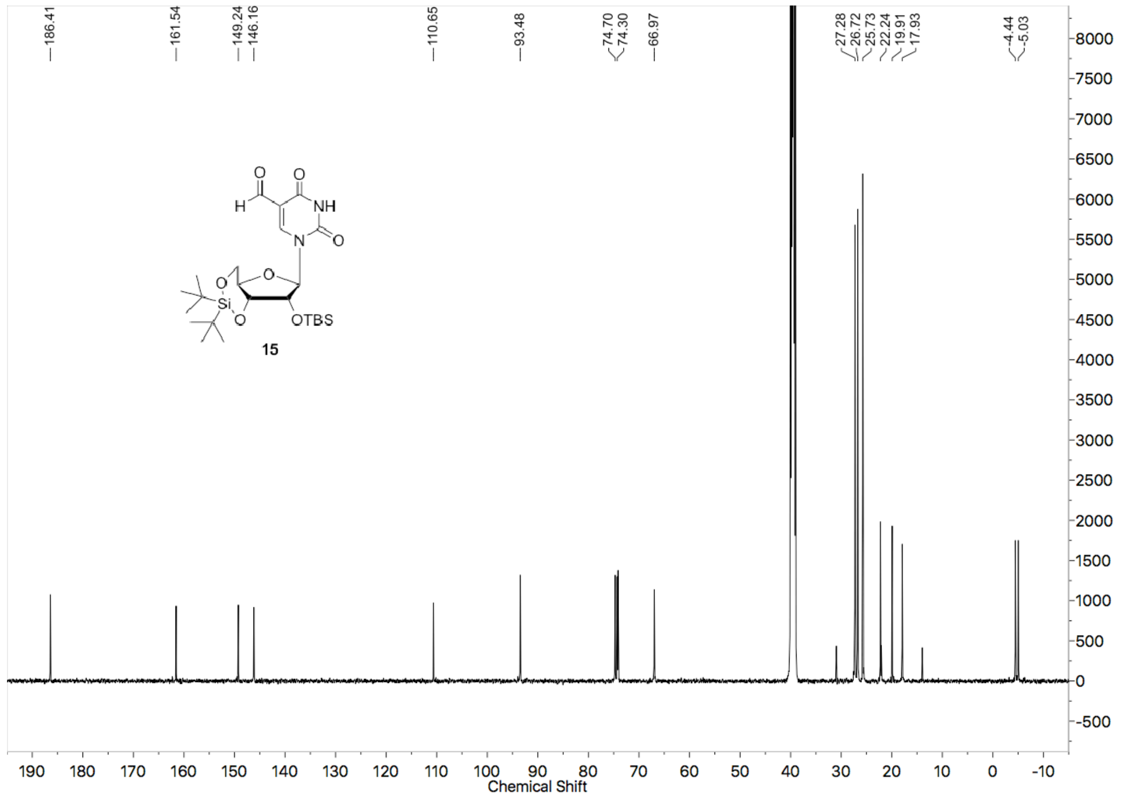

^{1}H NMR of compound **16** in DMSO-d$_6$

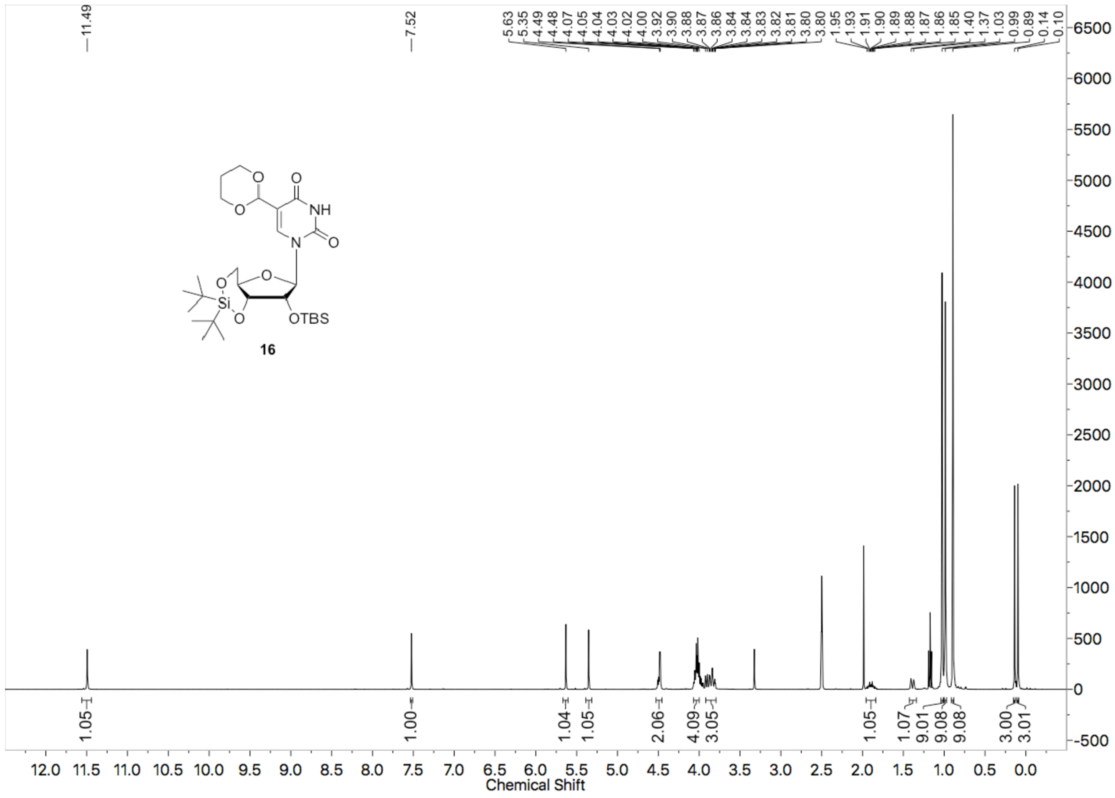

^{13}C NMR of compound **16** in DMSO-d$_6$

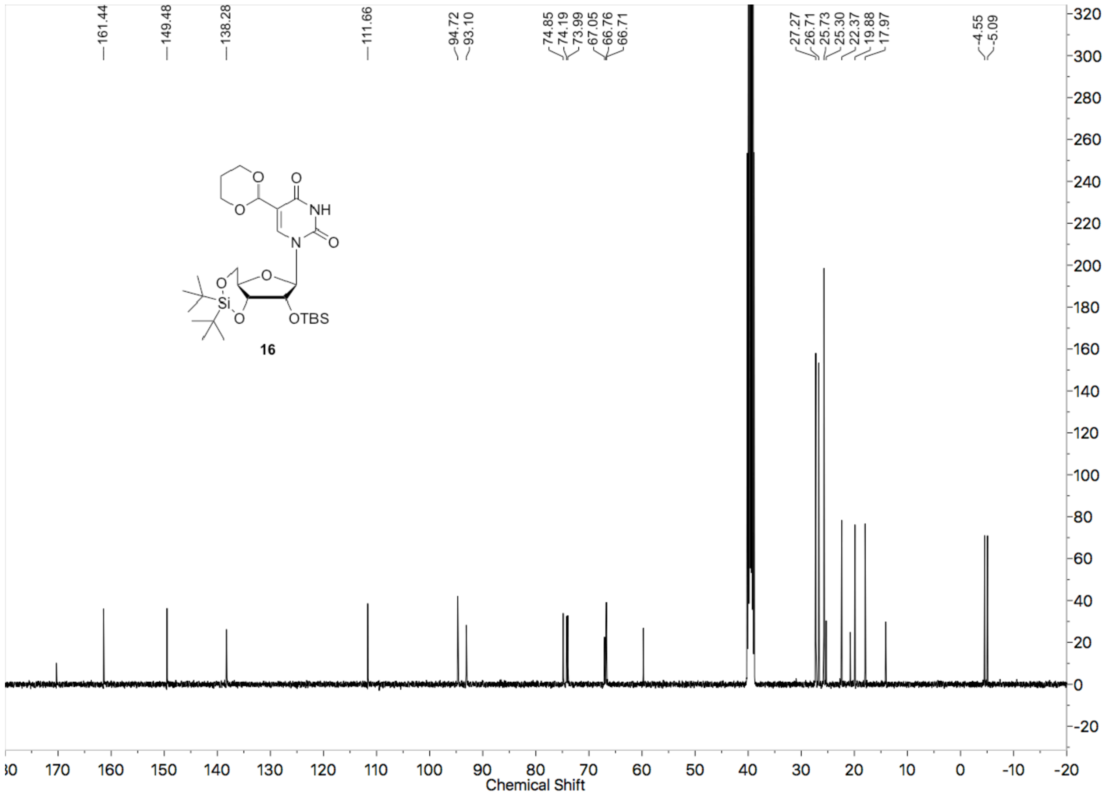

^{1}H NMR of compound **17** in DMSO-d$_6$

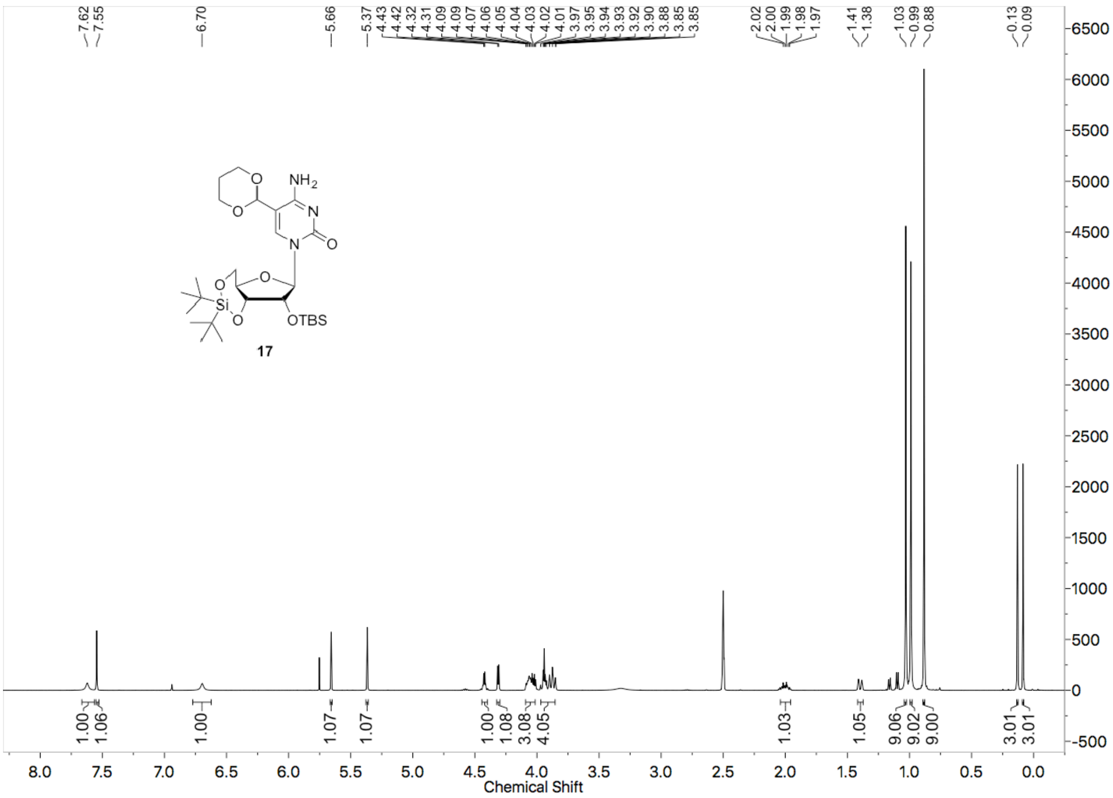

^{13}C NMR of compound **17** in DMSO-d$_6$

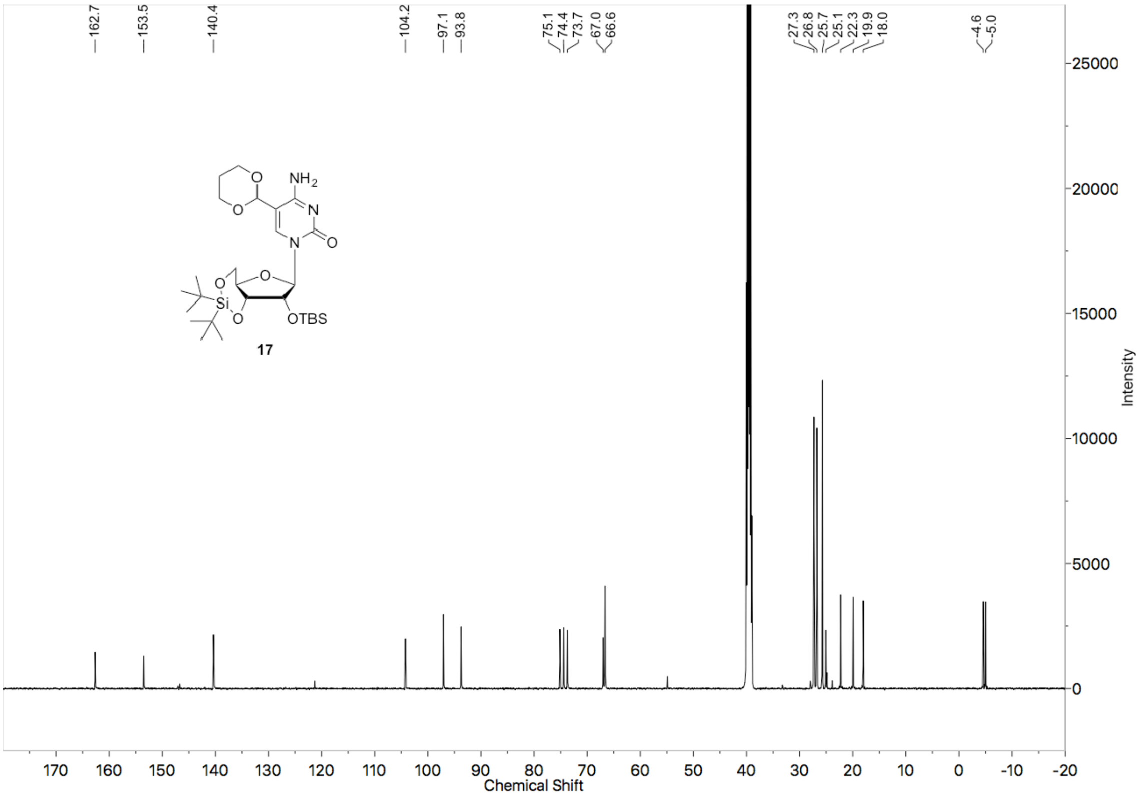

^{1}H NMR of compound **18** in DMSO-d_6

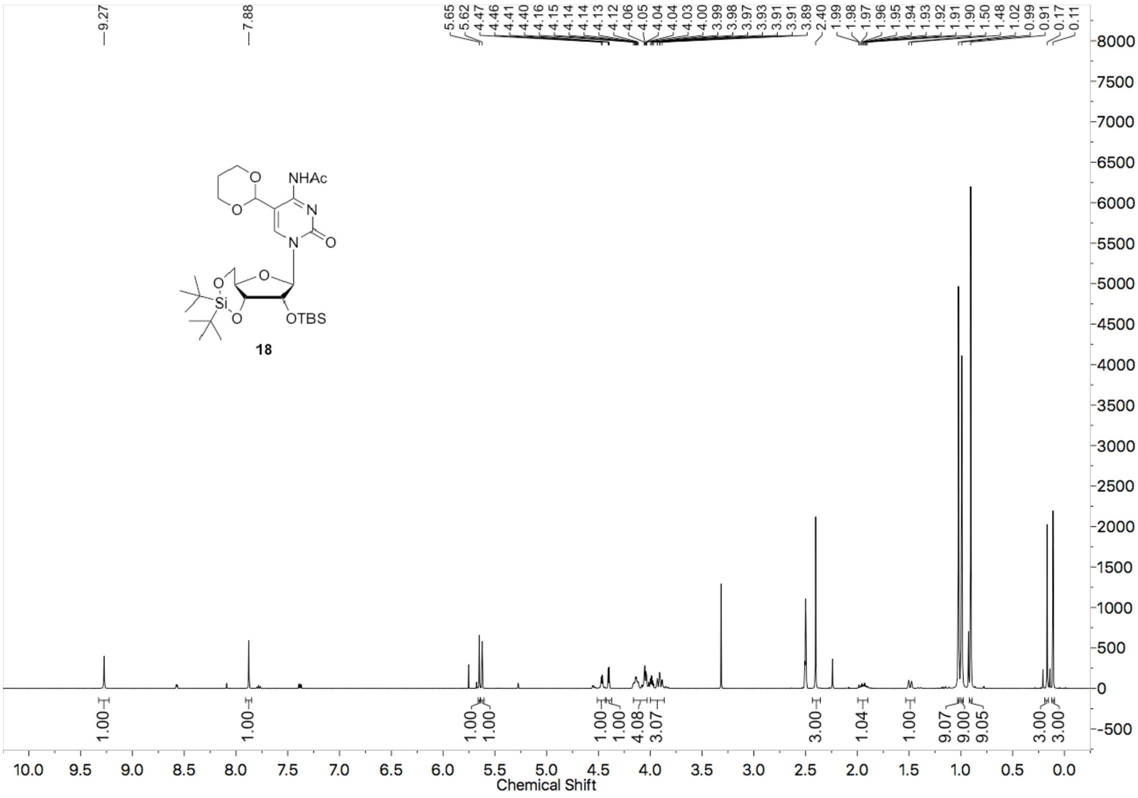

^{13}C NMR of compound **18** in DMSO-d_6

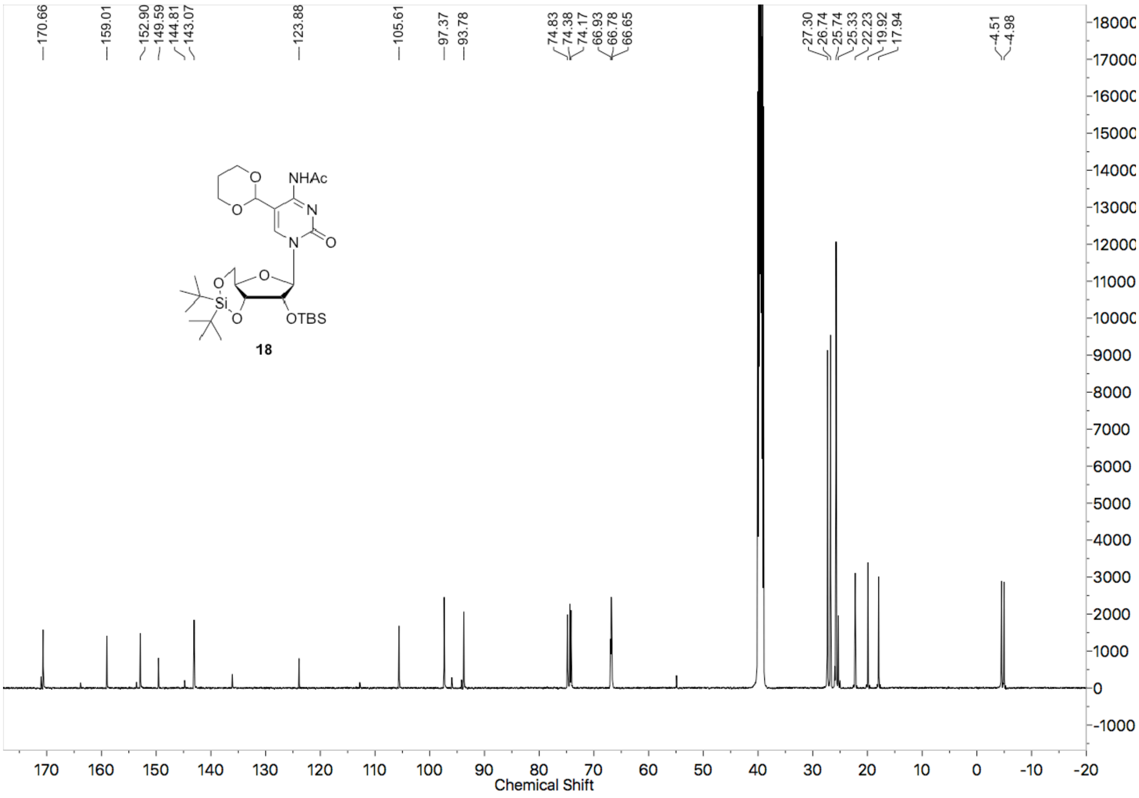

^{1}H NMR of compound **19** in DMSO-d_6

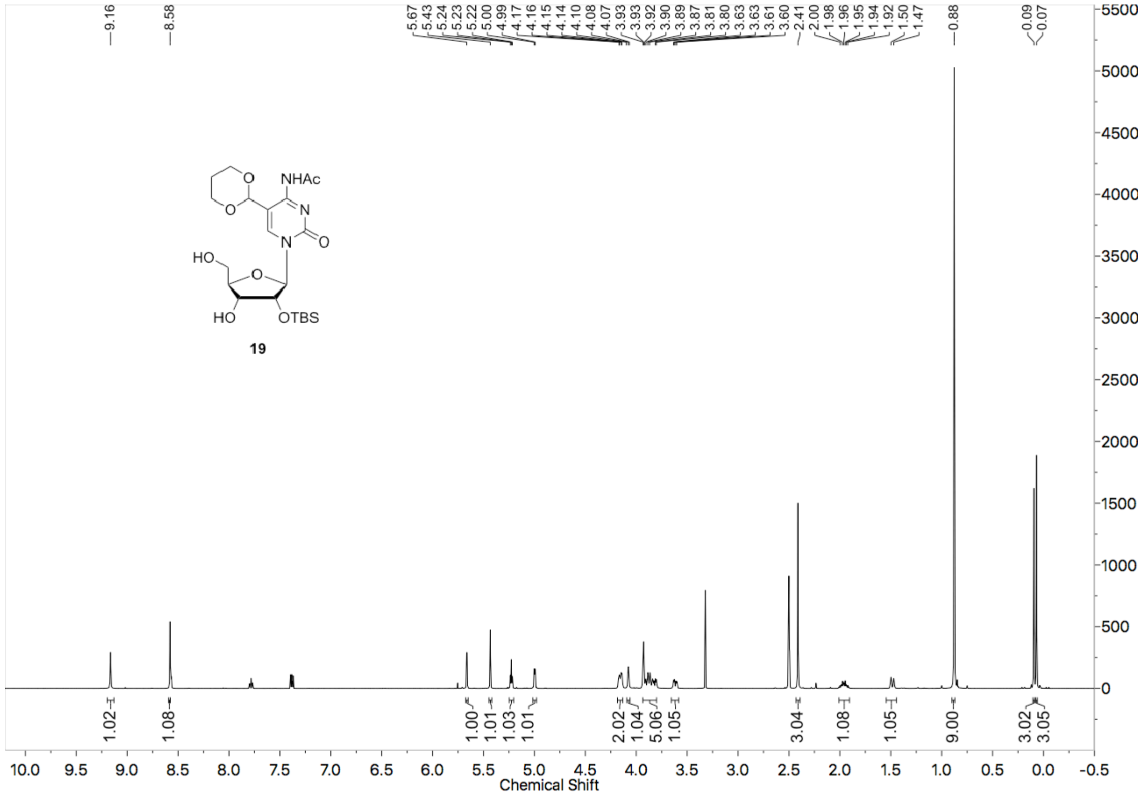

^{13}C NMR of compound **19** in DMSO-d_6

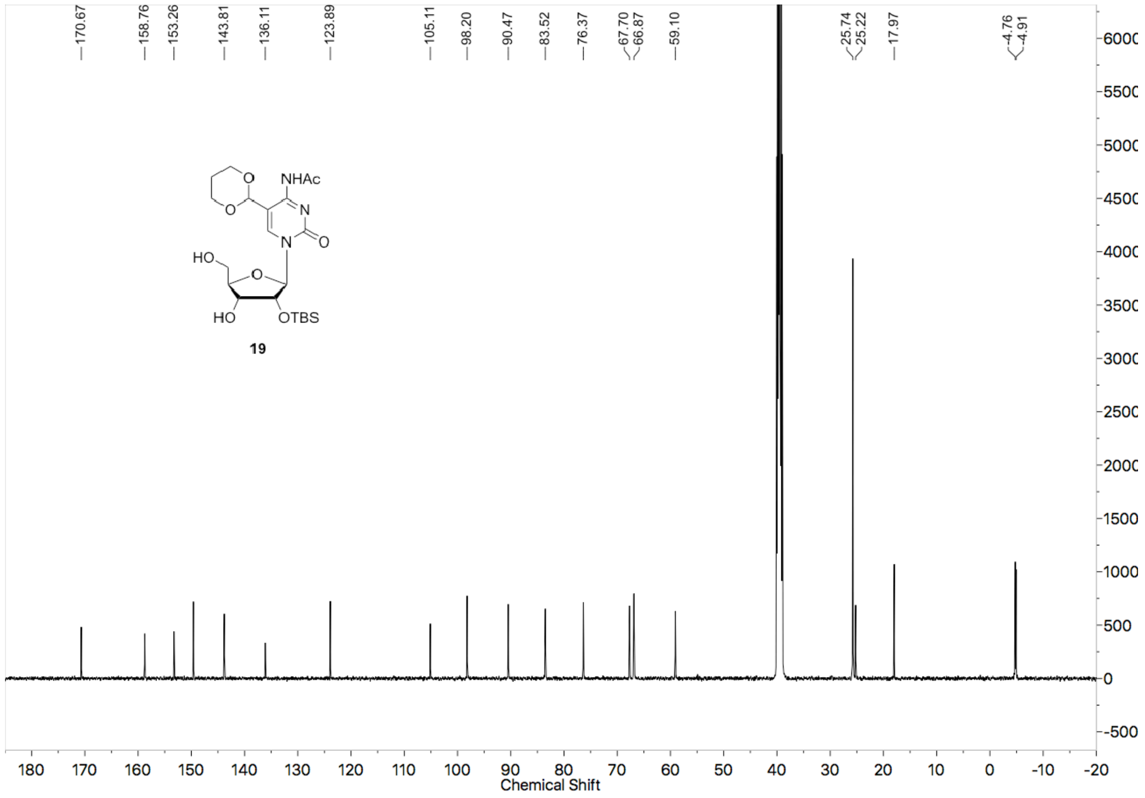

^{1}H NMR of compound **20** in DMSO-d$_6$

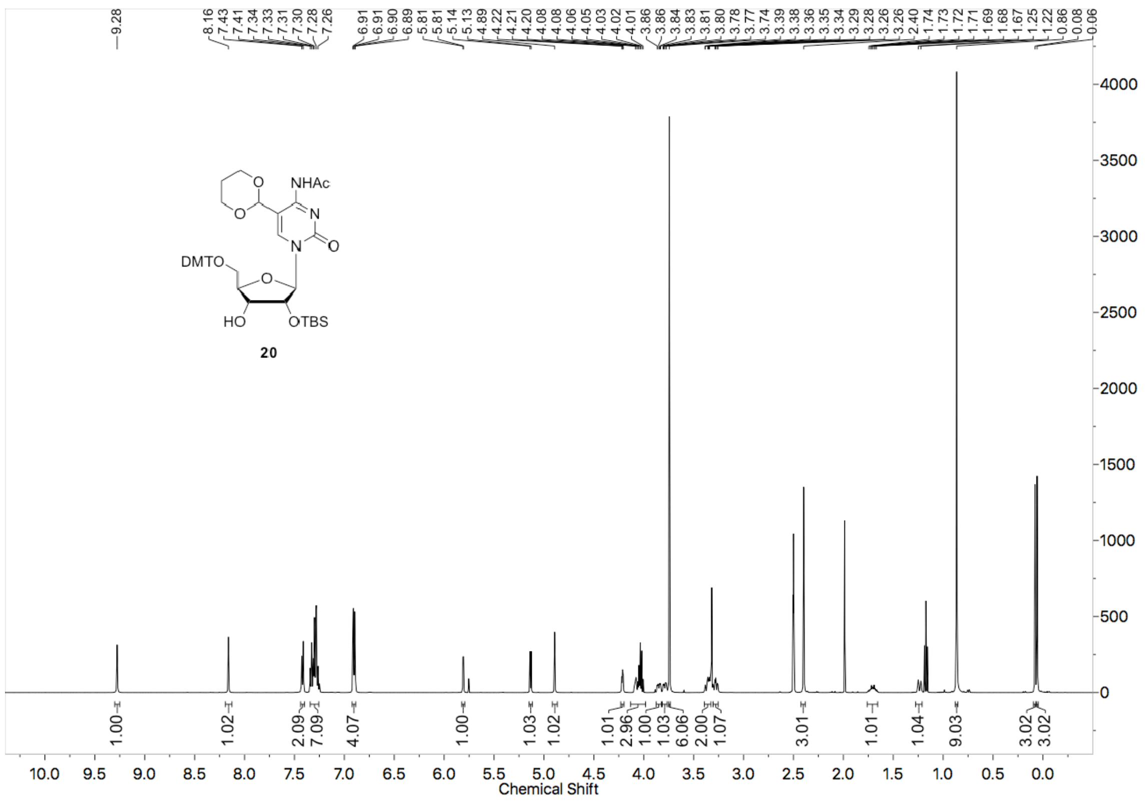

^{13}C NMR of compound **20** in DMSO-d$_6$

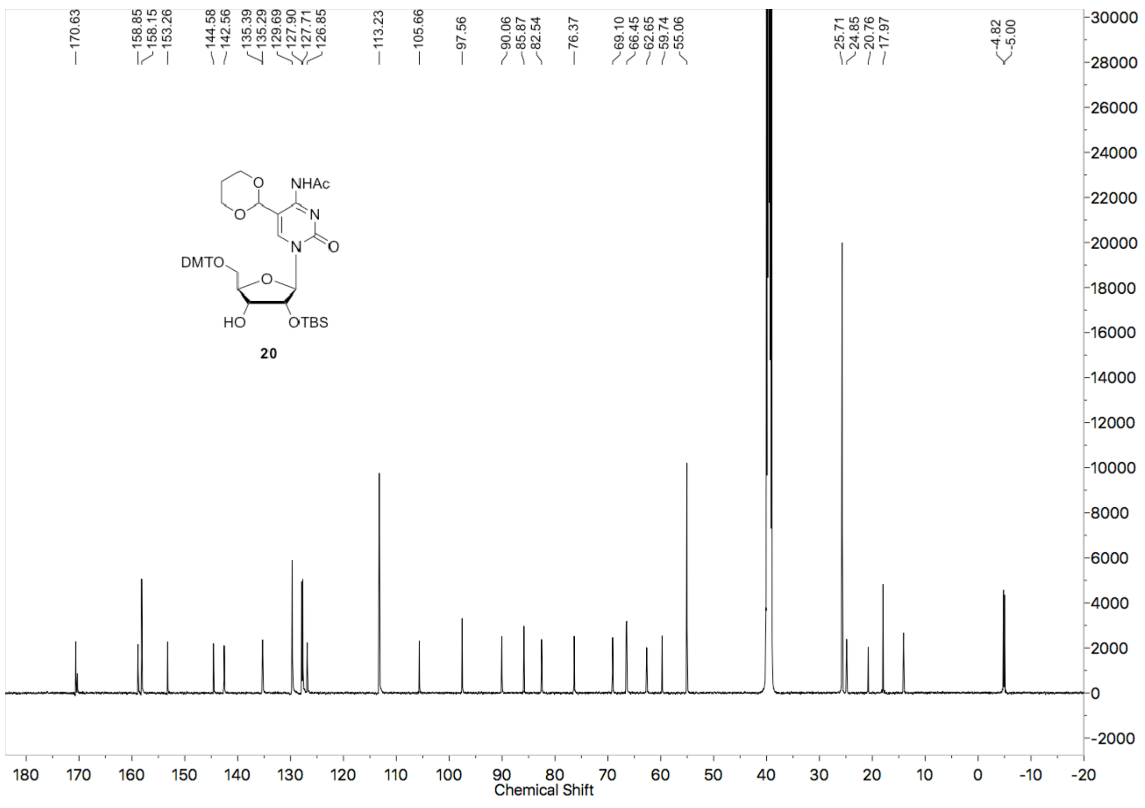

^{1}H NMR of phosphoramidite monomer **2** in DMSO-d_6

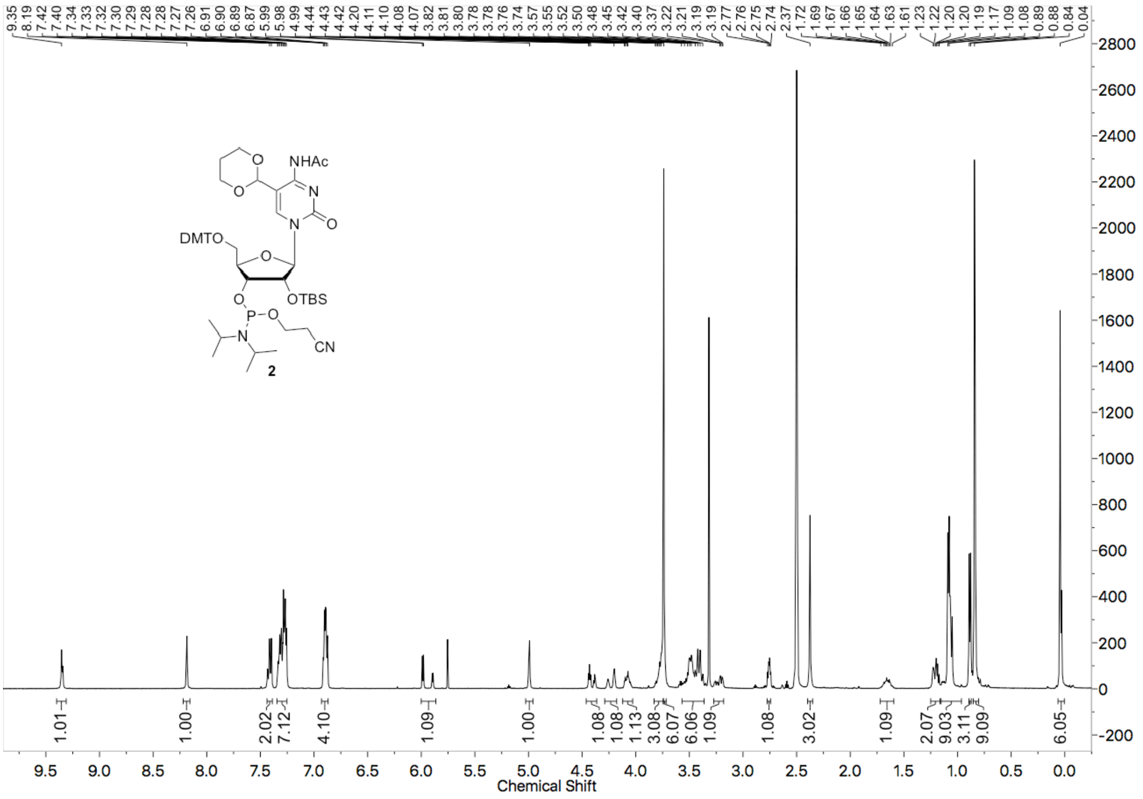

^{31}P NMR of phosphoramidite monomer **2** in DMSO-d_6

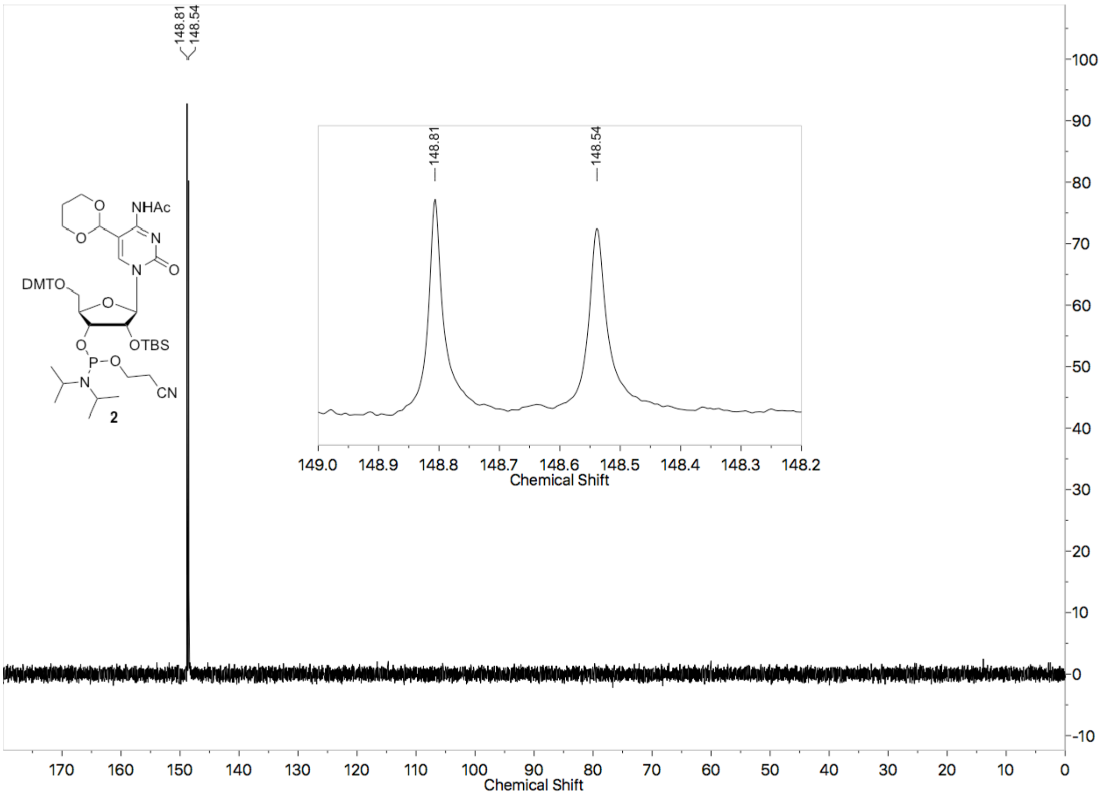

^{31}P NMR of phosphoramidite monomer **3** in DMSO-d_6

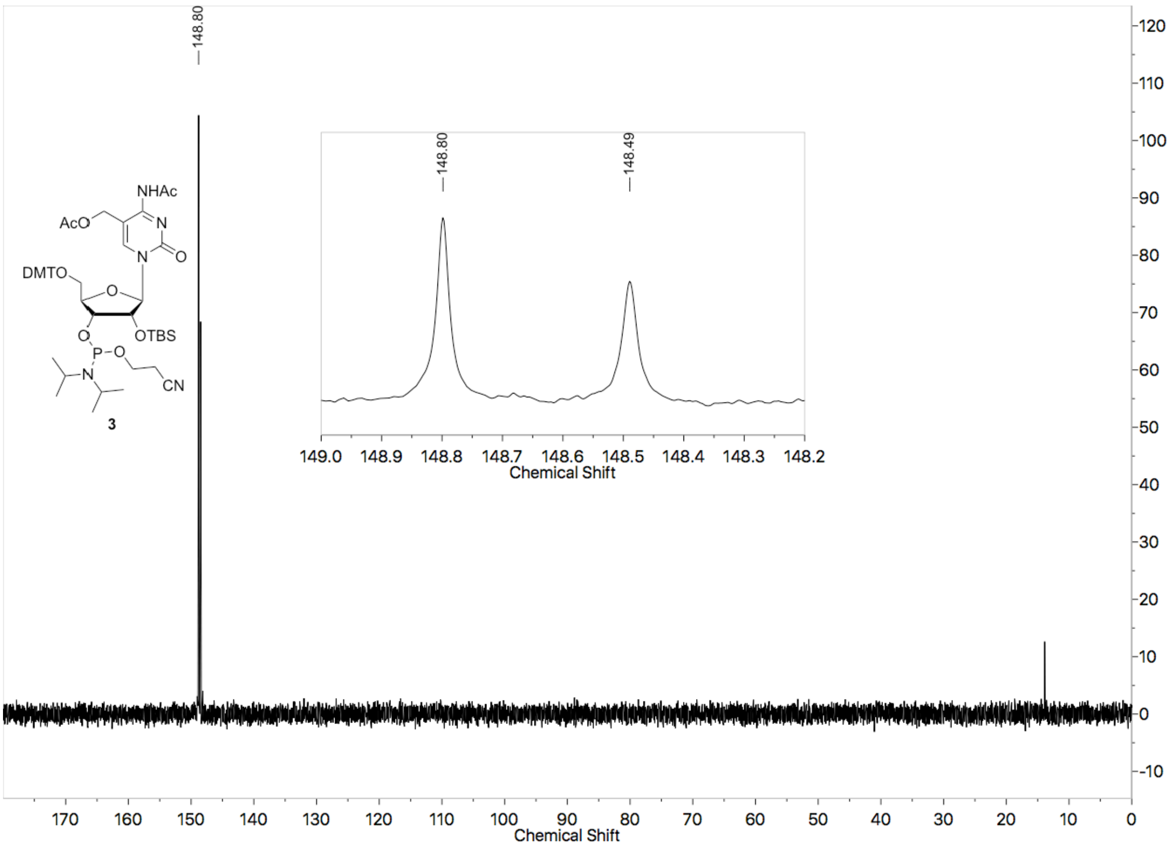

Supplement: Supplementary file 1 — Supplementary [file CBIC-18-2236-s001.pdf]
